# Targeting the CtBP1-FOXM1 transcriptional complex with small molecules to overcome MDR1-mediated chemoresistance in osteosarcoma cancer stem cells

**DOI:** 10.7150/jca.50255

**Published:** 2021-01-01

**Authors:** Xun Chen, Qian Zhang, Xiaoqian Dang, Tao Song, Yufei Wang, Zirui Yu, Shihui Zhang, Jinzhu Fan, Fei Cong, Wentao Zhang, Ning Duan

**Affiliations:** 1 Department of Orthopaedics, Honghui Hospital, Xi'an Jiaotong University, Xi'an 710054, Shaanxi, China.; 2 The department of surgery room, Xi'an Daxing Hospital, Xi'an 710016, Shaanxi, China.1Department of Orthopedics, the Second Affiliated Hospital of Xi'an Jiaotong University, Xi'an 710005, Shaanxi, China.; 3 Department of Orthopedics, the Second Affiliated Hospital of Xi'an Jiaotong University, Xi'an 710005, Shaanxi, China.

**Keywords:** CtBP1, FOXM1, MDR1, chemoresistance, osteosarcoma, NSM00158

## Abstract

Chemoresistance is a major barrier for the chemotherapy of osteosarcoma. The induction of multidrug resistance protein 1 (MDR1), an ATP-dependent transporter, can efflux anti-cancer drugs, thereby decreasing chemosensitivity. However, an actual involvement of MDR1 in the chemoresistance of osteosarcoma cells has not been established. We obtained two cisplatin (CDDP)-resistant osteosarcoma cancer stem cell (CSC) lines using sphere formation medium supplemented with CDDP. These two CDDP-resistant CSC cell lines showed substantial cell proliferation, colony formation, cell invasion, and *in vivo* tumor growth in the presence of CDDP. Microarray analysis revealed that three genes, *MDR1*, *FOXM1* (forkhead box M1), and *CtBP1* (C-Terminal binding protein 1), showed significant overexpression in both cell lines. Mechanistically, CtBP1 assembled with FOXM1 to form a transcriptional complex, which docked onto the *MDR1* promoter to activate *MDR1* expression. Knockdown or inhibition of the CtBP1-FOXM1 components with specific small molecules, including NSM00158 and NSC95397 for CtBP1 and RCM1 for FOXM1, significantly repressed *MDR1* expression. Administration of these three small molecules also significantly inhibited tumor growth in mouse tumor xenograft model. The MDR1-mediated chemoresistance could be reversed by NSM00158 and RCM1. Collectively, our data revealed that the CtBP1-FOXM1 complex activated *MDR1* expression and that targeting this complex with their specific inhibitors could reverse MDR1-mediated chemoresistance both *in vitro* and *in vivo*. Our results indicate a new therapeutic strategy for overcoming chemoresistance during osteosarcoma treatment.

## Introduction

Osteosarcoma is the most common malignant tumor arising in the bones of adolescents 1. As observed with other types of tumors, therapeutic strategies for osteosarcoma are mainly divided into three types: surgical resection of the primary lesion, chemotherapy, and radiotherapy 2. The chemotherapeutic drugs used most often to treat osteosarcoma include cisplatin (CDDP), doxorubicin, and methotrexate (MTX) 2,3. Osteosarcoma patients, especially those with metastasis, often develop chemoresistance following a period of treatment 2-4. This chemoresistance is largely due to the presence of cancer stem cells (CSCs), a class of cells capable of self-renewal and differentiation that show resistance to most chemotherapeutic agents 5,6. The underlying mechanisms of CSC-mediated chemoresistance in osteosarcoma are complicated but mainly include insufficient intracellular drug accumulation; dysregulation of genes, microRNAs and long non-coding RNAs; and inactivation of apoptotic signaling pathways 5,6.

Multidrug resistance protein 1 (MDR1), also known as ATP-binding cassette subfamily B member 1 (ABCB1), is a transporter P-glycoprotein located on the cell membrane 7,8. Increased level of MDR1 has been reported to function in the chemoresistance of a variety of cancer types, as the protein can serve as an efflux transporter for many chemotherapeutic drugs, including doxorubicin, CDDP, and MTX 7,8. The mechanisms underlying MDR1 accumulation have been intensively investigated. At the transcriptional level, several transcription factors, including NF-κB (nuclear factor-kappa B), p53, and YBX1 (Y-box binding protein 1), can directly bind to the *MDR1* promoter to activate its expression 9,10. In addition to these transcription factors, many cell signaling pathways, such as Wnt/β-catenin pathway, PI3K/AKT (phosphoinositide-3-kinase/AKT serine/threonine kinase 1) pathway, MAPK/ERK (mitogen-activated protein kinase 1/extracellular-signal-regulated kinase) pathway, and p38 MAPK pathway, are also involved in the regulation of *MDR1* expression 11,12. MDR1 overexpression has been observed in human osteosarcoma doxorubicin-resistant cell lines by at least two groups around the world. For example, Ye et al. found that NVP‐TAE684, a kinase inhibitor, could inhibit MDR1 function and reverse MDR1-mediated chemoresistance in osteosarcoma 13. Using the same doxorubicin-resistant cell lines, Wang and colleagues demonstrated that the transcription factor STAT3 (signal transducer and activator of transcription 3) could activate *MDR1* expression and that attenuation of STAT3 phosphorylation induced apoptosis and increased chemosensitivity 14.

Two human multidrug resistant cancer cell lines, NCI/ADR-RES and A2780/DX, show activation of *MDR1* by the transcriptional regulator CtBP1 (C-Terminal binding protein 1) 15. However, the mechanism by which CtBP1 activates *MDR1* in this process is not yet understood. CtBP1 can mediate gene expression by serving as either a transcriptional corepressor or a coactivator 16. CtBP1 overexpression is observed in multiple cancer types, such as melanoma, osteosarcoma, colon cancer, and prostate cancer 16. In these cancers, overexpression of CtBP1 can cause the suppression of multiple genes involved in genome instability (e.g., *BRCA1* [breast cancer 1 and 2]), apoptosis (e.g., *BAX* [BCL2 associated X], *BIK* [BCL2 interacting killer], *BIM* [BCL2 interacting mediator], *PUMA* [p53 upregulated modulator of apoptosis], and *PERP* [p53 apoptosis effector related to PMP22]), cell proliferation/migration/invasion (e.g., *PTEN* [phosphatase and tensin homolog], *CDKN1A* [cyclin dependent kinase inhibitor 1A], and *CDKN2A*), and the epithelial-mesenchymal transition (EMT) (e.g., *CDH1* [cadherin 1], also known as E-cadherin) 16.

CtBP1 has a conservative working mechanism in these processes, whereby it interacts with transcription factors or transcriptional repressors/activators through a conserved PXDLS motif (where X represents any amino acid) 16. A biochemical study of CtBP1 proteins with constructed point mutations of this motif showed that only the P, D, and L amino acids are necessary for these interactions 17. In addition to serving as a corepressor, CtBP1 also has a transcriptional activation role in gene expression. In gastrointestinal endocrine cells, CtBP1 transactivates the expression of *NEUROD1* (neuronal differentiation 1) by assembling a complex with the transcription factor RREB1 (RAS-responsive element binding protein 1), a histone modification enzyme LSD1 (lysine demethylase 1), and a histone acetyltransferase p300 associated protein PCAF (P300/CBP-associated factor) 18. In human keratinocytes, CtBP1 can activate the expression of several epidermal differentiation genes, including* PKP1* (plakophilin 1), *DLX5* (distal-less homeobox 5), and *PPL* (periplakin), by assembling a complex with two transcription factors, ZNF750 (zinc finger protein 750) and KLF4 (kruppel-like factor 4), and a transcriptional corepressor RCOR1 (REST corepressor 1) 19.

The important roles of CtBP1 in mediating gene expression have suggested its potential therapeutic role as a target in different disease processes 16. Several small molecules, including NSC95397, MTOB (4-methylthio-2-oxobutanoate), phenylpyruvate, and 2-hydroxyimino-3-phenylproanoic acid, as well as the peptide CP61 (cyclic peptide-61), have been identified as inhibitors of CtBP1 transcriptional activity 16. Most recently, our group also identified a small molecule NSM00158 that could specifically inhibit CtBP2 function 20. The administration of NSM00158 in a mouse bone fracture model prevented the occurrence of nonunion after bone fracture by reversing CtBP2-mediated transrepression 20. CtBP1 and CtBP2 are highly conserved homologues that share over 80% amino acid identity 20. Importantly, they also have similar interaction modes with other proteins through the PXDLS motif.

In our clinical treatment, we often observe that osteosarcoma patients develop resistance to chemotherapy. Here, we investigated the underlying mechanism for CSC-mediated chemoresistance using two CDDP-resistant CSC cell lines in the MG63 osteosarcoma cell background. Microarray analysis revealed that *MDR1*, *CtBP1,* and *FOXM1* **(**forkhead box M1) were significantly overexpressed in CDDP-resistant CSC cell lines. Subsequent *in vitro* and *in vivo* experiments demonstrated that FOXM1 could recruit CtBP1 to the *MDR1* promoter and that CtBP1 acted as an activator to induce the expression of *MDR1*. We also used *in vitro* and *in vivo* experiments to examine whether two CtBP1 inhibitors (NSC95397 and NSM00158) and one FOXM1 inhibitor (RCM1) could decrease *MDR1* expression and inhibit *in vitro* cell proliferation, colony formation, cell invasion, and *in vivo* tumor growth. Our results suggest that targeting the CtBP1-FOXM1 complex could significantly reverse MDR1-mediated chemoresistance in osteosarcoma.

## Materials and methods

### Cell lines and cell culture

The human osteosarcoma cell line MG63 was purchased from the American Type Culture Collection (ATCC) (Manassas, VA, USA, #CRL-1427) and grown in Dulbecco's Modified Eagle Medium (DMEM) (Sigma-Aldrich, Shanghai, China, #D5796) containing 10% fetal bovine serum (FBS) (Sigma-Aldrich, #12003C), and 100 U·mL^-1^ penicillin-streptomycin (Sigma-Aldrich, #P4458). Using MG63 as a maternal cell line, two CDDP-resistant cell lines (#R1 and #R2) were obtained by tumor sphere formation assay. Briefly, MG63 cells were grown in stem cell medium containing DMEM/F12 (Sigma-Aldrich, #51445C), 1×B27 (Thermo Fisher Scientific, Shanghai, China, #A3653401), 20 ng/mL epidermal growth factor (EGF) (Thermo Fisher Scientific, #PHG0313), 20 ng/mL basic fibroblast growth factor (bFGF) (Sigma-Aldrich, #F0291), 4 μg/mL heparin (Sigma-Aldrich, #E4643), and 50 μM CDDP. Cells were seeded into medium at a density of approximately 1000 cells/mL and incubated at 37°C for 14 days, with a medium change every three days. The formed individual spheres were separately collected and enzymatically dissociated with Accutase^TM^ cell detachment solution (Sigma-Aldrich, #SCR005) at room temperature for 10 min. Single-cell suspensions were used for the required experiments.

### Vector construction

A 1500-bp length of *MDR1* promoter was cloned into pGL4.26 luciferase vector using KpnI and XhoI sites. The generated pGL4.26-pMDR1^WT^ was used as a template to create its mutant vector in which the FOXM1 binding site GTAAACAA was mutated to GGTTTATT. The coding sequence of *CtBP1* was cloned into pGADT7 empty vector using EcoRI and BamHI sites. Full-length coding sequences of *FOXM1* and its mutant (*FOXM1*^△PLDLI^) were cloned into pGBKT7 empty vector using EcoRI and BamHI sites. Full-length coding sequences of *FOXM1* and *FOXM1*^△PLDLI^ were cloned into pCDNA3-MYC empty vector using HindIII and EcoRI sites. Full-length coding sequence of *CtBP1* was cloned into pCDNA3-2×Flag empty vector using HindIII and EcoRI sites. All primers used for vector constructions were listed in Supplementary Table 1.

### Cell transfection

The MG63-R1 and MG63-R2 cells were used for knocking down either *CtBP1* or* FOXM1* with their corresponding shRNA lentiviral transduction particles, including #TRCN0000285086 for *CtBP1* and #TRCN0000273939 for *FOXM1*, purchased from Sigma-Aldrich. These two particles and a control particle containing pLKO.1 empty vector were individually transfected into MG63-R1 and MG63-R2 cells with FuGene 6 (Roche Diagnostics Corp., Indianapolis, IN, USA, #E2691), according to the manufacturers' protocol. After incubation at 37°C for 12 h, the transfected cells were cultured in DMEM medium containing 1 μg/mL puromycin for selection. Single puromycin-resistant cells were collected for use in experiments.

### Immunofluorescent staining

The MG63, MG63-R1, and MG63-R2 cells were fixed in 4% paraformaldehyde (Sigma-Aldrich, #158127), followed by blocking with 5% bovine serum albumin (BSA) (Sigma-Aldrich, #A2153). The anti-human CD133 antibody (1:50 dilution, Sigma-Aldrich, #MAB4399-I) was added and incubated at 4 °C for 12 h. After washing three times with phosphate-buffered saline (PBS), cells were counterstained with 4′,6-diamidino-2-phenylindole (DAPI) (Sigma-Aldrich, #D9542). Images were captured using a fluorescence microscope (United Scope LLC, #ZM-4TW3-FOR-9M).

### Western blotting

Cells were washed twice with PBS and lysed with 1 × RIPA buffer (Sigma-Aldrich, #R0278) containing a protease inhibitor cocktail (Sigma-Aldrich, P8340). Equal amounts of total protein (approximately 50 μg) were resolved in 10% SDS-PAGE gels and transferred to PVDF membranes, followed by blocking with 5% skim milk powder dissolved in phosphate buffered saline-Tween20 (PBST) for 1 h at room temperature. Membranes were probed with the following primary antibodies: anti-CD133 (Sigma-Aldrich, #MAB4399-I), anti-CtBP1 (BD Bioscience, San Jose, CA, USA, #612042), anti-FOXM1 (Sigma-Aldrich, #AV39518), anti-MDR1 (Thermo Fisher Scientific, #PA5-28801), and anti-GAPDH (Thermo Fisher Scientific, #MA5-15738-BTIN). After incubating with primary antibodies at 4°C overnight, membranes were washed five times with PBST buffer and then probed with peroxidase-conjugated secondary antibodies (Abcam, Cambridge, UK; mouse, #ab205719; and rabbit, #ab205718) for 1 h at room temperature. The protein signals were visualized using an ECL detection reagent (Sigma-Aldrich, #GERPN2109).

### Total RNA extraction, microarray analysis, and real-time quantitative PCR (RT-qPCR)

Cells under 80% confluency were used for isolation of total RNA with an RNeasy plus kit (Qiagen, Hilden, Germany, #74134). For microarrays, 1 µg of total RNA was used to detect aberrantly expressed genes with a human GE 4×44K v2 microarray kit (Agilent Technologies, Santa Clara, CA, USA, #G4845A), following a previous protocol 21. For RT-qPCR analyses, 1 µg of total RNA was used for reverse transcription to synthesize cDNA with a PrimeScript RT reagent kit (Takara, Beijing, China, #RR0378). After diluting 20-fold, cDNAs were subjected to RT-qPCR using a One-step Green PrimeScript RT-PCR kit (Takara, #RR086B) with primers included in Supplementary Table-2. Each sample was run in triplicate and gene expression levels were normalized to β-Actin according to the 2^-∆∆Ct^ method. PCR procedures were as follows: 95 °C for 5 min, followed by 40 cycles of 95 °C for 10 seconds and 68 °C for 40 seconds.

### Cell proliferation, colony formation, and cell migration assays

Cell proliferation was determined using an MTT kit (Abcam, #ab211091) according the protocol provided by the manufacturer. Briefly, cells were seeded into 96-well plates with a density of 1×10^3^/well. After culturing at 37°C for 4 h (0 day), or 1, 2, 3, 4, and 5 days, cells in each well were incubated with 20 μL MTT reagent. The microplates were further incubated at 37°C for 4 h and cells were dissolved with acid-isopropanol. The absorbance was measured at OD_590_ nm. For colony formation assay, cells (~500) were plated into 6-well plates and maintained in DMEM medium for 14 days, with a medium change every three days. The colonies were washed twice with PBS buffer, fixed with 4% paraformaldehyde, and stained with 0.1% crystal violet (Sigma-Aldrich, #C0775). The colonies were photographed, and colony numbers were counted manually. Cell migration assays were performed using the Boyden Chamber assay following a previous protocol 22. Briefly, a cell suspension in serum-free DMEM medium was loaded into the upper insert of the Boyden chamber (Sigma-Aldrich, #ECM550). The lower inserts were filled with DMEM medium containing 10% FBS. The entire chamber was incubated at 37°C for 24 h, and cells on the lower chambers were fixed with 4% paraformaldehyde, followed by staining with 0.1% crystal violet. The invading cells were photographed, and colony numbers were counted manually.

### Drug treatment

Cells at 80% confluency were washed twice with PBS buffer, followed by treatments with 2 µM NSM00158, 20 µM NSC95397 (Sigma-Aldrich, #N1786), or 1 µM RCM1 (Sigma-Aldrich, #SML2625). Cells were further incubated at 37°C for 6 h, and then were collected and used in experiments.

### Chromatin immunoprecipitation (ChIP) assay

ChIP assays were performed using the Millipore ChIP Assay Kit (Millipore, Burlington, MA, USA, #17295), as described previously 21. Protein-G-magnetic-beads (20 μl) (Abcam, #ab214286) were coupled to 2.5 μL anti-FOXM1, anti-CtBP1, or IgG antibody, followed by incubating with cell lysates. The purified DNA was digested with proteinase K (Sigma-Aldrich, #3115887001) and then subjected to RT-qPCR analysis with the following primers: forward, 5'-ACTCCTTCCTTCAATTTGTGC-3', reverse, TACCATATGATATTTCAAACA. The relative occupancies of CtBP1 and FOXM1 on the *MDR1* promoter were normalized using output/input.

### Luciferase activity assay

Luciferase assays were performed using the Promega Dual-luciferase Reporter Assay System (Promega, Madison, WI, USA, #E1910), as described previously 20. Briefly, the MG63, MG63-R1, and MG63-R2 cells were co-transfected pGL4.26-pMDR1^WT^ + Renilla or pGL4.26-pMDR1^Mut^ + Renilla. The transfected cells were cultured at 37°C for 24 h, followed by cell collection and luciferase assays.

### Immunoprecipitation (IP) and co-immunoprecipitation (Co-IP) assays

Equal numbers of MG63, MG63-R1, and MG63-R2 cells (approximately 2×10^7^ cells) were lysed in 3 mL RIPA buffer containing protease inhibitor cocktail. After centrifuging at 13,000 rpm for 15 min, 0.2 mL of supernatant was used as input and the other supernatant was subjected to IP assays using Protein-G magnetic beads coupled with anti-FOXM1, anti-CtBP1, or IgG antibodies. The input and purified protein complexes subjected to western blotting to detect FOXM1 and CtBP1 protein levels. For Co-IP assay, MG63 cells were transfected with different combinations of Flag-tagged and MYC-tagged plasmids, as indicated in the figures. Equal amounts of the transfected cells (approximately 2×10^7^ cells) were subjected to IP procedures using Flag-agarose beads (Sigma-Aldrich, #A4596) and MYC-agarose beads (Sigma-Aldrich, #A7470). The input and purified protein complexes were subjected to western blotting using anti-Flag and anti-MYC antibodies.

### Protein interaction in yeast cells

The AH109 yeast cells were co-transformed with the following combinations of plasmids: pGADT7+pGBKT7, pGADT7+pGBKT7-FOXM1, pGADT7+pGBKT7-FOXM1^△PLDLI^, pGADT7-CtBP1+pGBKT7, pGADT7-CtBP1+pGBKT7-FOXM1, and pGADT7-CtBP1+pGBKT7-FOXM1^△PLDLI^. The transformed cells were selected in synthetic complete medium lacking Trp and Leu (SC-TL). The positive colonies were further grown in synthetic complete medium lacking His, Trp, and Leu (SC-HTL) to determine protein interactions. The β-galactosidase activity was measured following a protocol described previously 23.

### Tumor xenograft model

Equal volumes of suspensions of MG63, MG63-R1, MG63-R1-CtBP1-KD1, MG63-R1-FOXM1-KD1, MG63-R2, MG63-R2-CtBP1-KD1, and MG63-R2-FOXM1-KD1 cells were injected subcutaneously into six-week-old C57BL/6 mice (n=10 for each cell line). The tumor width and length were measured at 5-day intervals and tumor volumes were determined using the formula: volume = (length × width^2^)/2. The effects of NSM00158, NSC95397, and RCM1 on inhibition of tumor growth were examined by subcutaneous injection of equal volumes of MG63 and MG63-R1 cell suspensions into six-week-old C57BL/6 mice (n=50 for each cell line). Small molecules were injected into mice (n=8 for each small molecule) at different dosages (NSM00158: 2 mg/kg; NSC95397: 6 mg/kg; and RCM1: 1.7 mg/kg) every five days when the tumor volumes had reached approximately 200 mm^3^. Tumor volumes were also determined at 5-day intervals. The effects of NSM00158, RCM1, CDDP, NSM00158+CDDP, and RCM1+CDDP were evaluated by subcutaneous injection of MG63-R1 cell suspension into six-week-old C57BL/6 mice (n=50). CDDP (5 mg/kg) was combined with or without NSM00158 (2 mg/kg) and RCM1 (1.7 mg/kg), followed by injecting into mice (n=8 for each small molecule) when tumor volumes had reached approximately 200 mm^3^. The tumor volumes were determined at 5-day intervals. All animal experiments were performed following a protocol reviewed by the Institutional Animal Care and Use Committee (IACUC) of Honghui Hospital in Xi'an Jiao Tong University.

### Statistical analysis

All experiments were independently performed in triplicate, and statistical analyses were performed using SPSS (Statistical Package for Social Sciences) software (version 26, IBM, USA). Data are presented as the mean ± standard deviation (SD), with statistical significance defined as *P* < 0.05 (*), *P* < 0.01 (**) and *P* < 0.001 (***). Figures were presented using the Prism-GraphPad software (version 8).

## Results

### MG63-R1 and MG63-R2 cells possessed CSC properties and CDDP resistance

**Figure 1:**
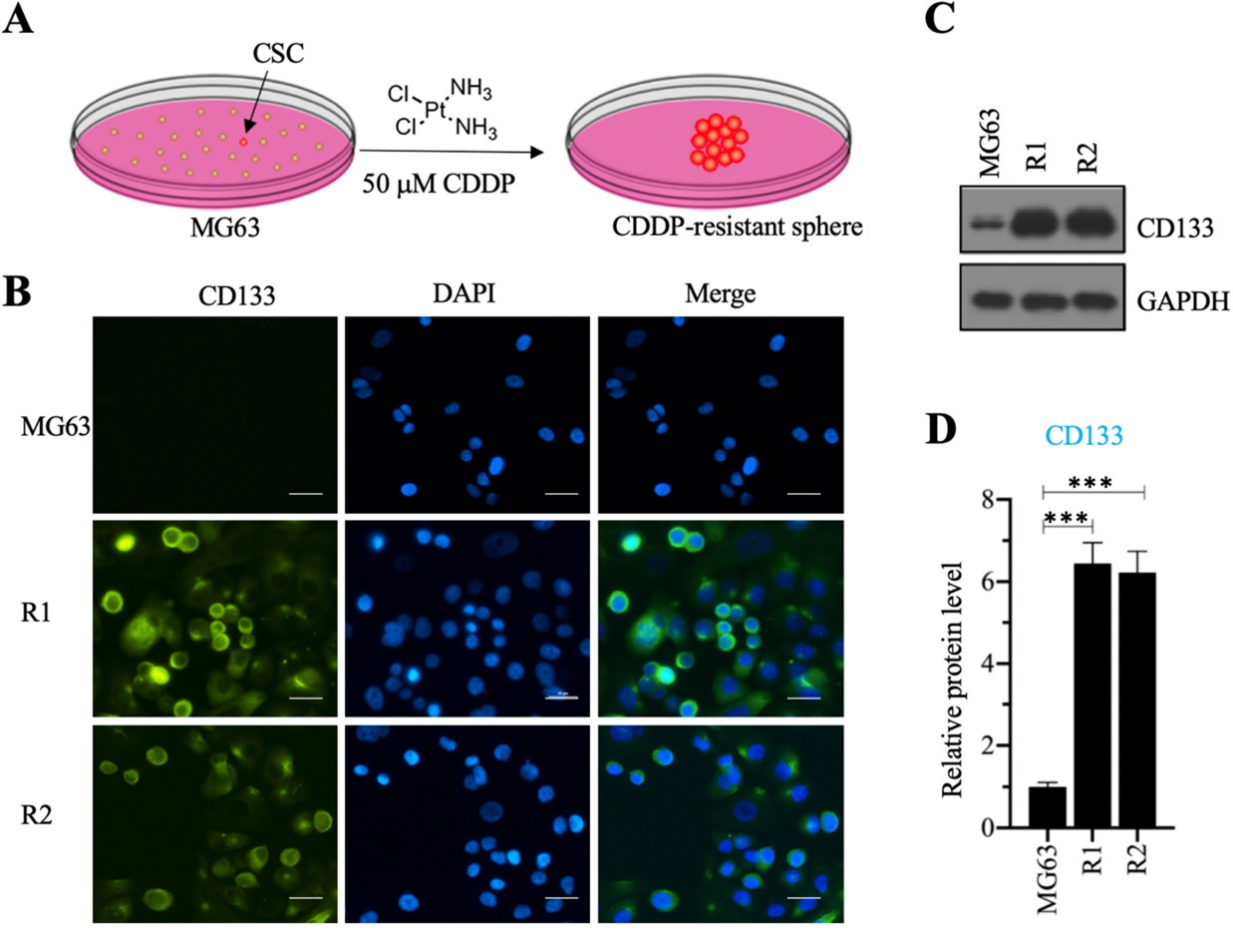
** Screening cisplatin (CDDP)-resistant osteosarcoma cancer stem cells (CSCs) and detecting CD133 level in these cells.** (A) A schematic diagram of the screening procedure for CDDP-resistant osteosarcoma CSCs. (B) Immunofluorescence staining for CD133. An osteosarcoma CSC marker CD133 was stained using its specific antibody in MG63, MG63-R1, and MG63-R2 cells. The same cells were also counterstained with DAPI. Bars=25 µm. (C and D) Protein level of CD133. Total cell lysates from MG63, MG63-R1, and MG63-R2 cells were subjected to western blotting to determine CD133 and GAPDH (loading control) levels (C). The CD133 protein signals in different cells were quantified and normalized to their corresponding GAPDH (D). *** *P* < 0.001.

We investigated the roles of CSC in the osteosarcoma chemoresistance by initially screening osteosarcoma CSCs using MG63 as a maternal cell line. We cultured MG63 cells in sphere formation medium supplemented with 50 µM CDDP for two weeks and obtained two CDDP-resistant spheres (Figure 1A), designated as MG63-R1 and MG63-R2, which were collected separately after enzymatic dissociation with Accutase™ cell detachment solution. The two cell lines were confirmed to possess CSC properties by immunofluorescence staining using the osteosarcoma CSC marker CD133 (Figure 1B), whereas the MG63 maternal cell line showed no CD133 positivity. Western blotting assays confirmed significant expression of CD133 protein in both the MG63-R1 and the MG63-R2 cell lines (Figures 1C and 1D).

**Figure 2:**
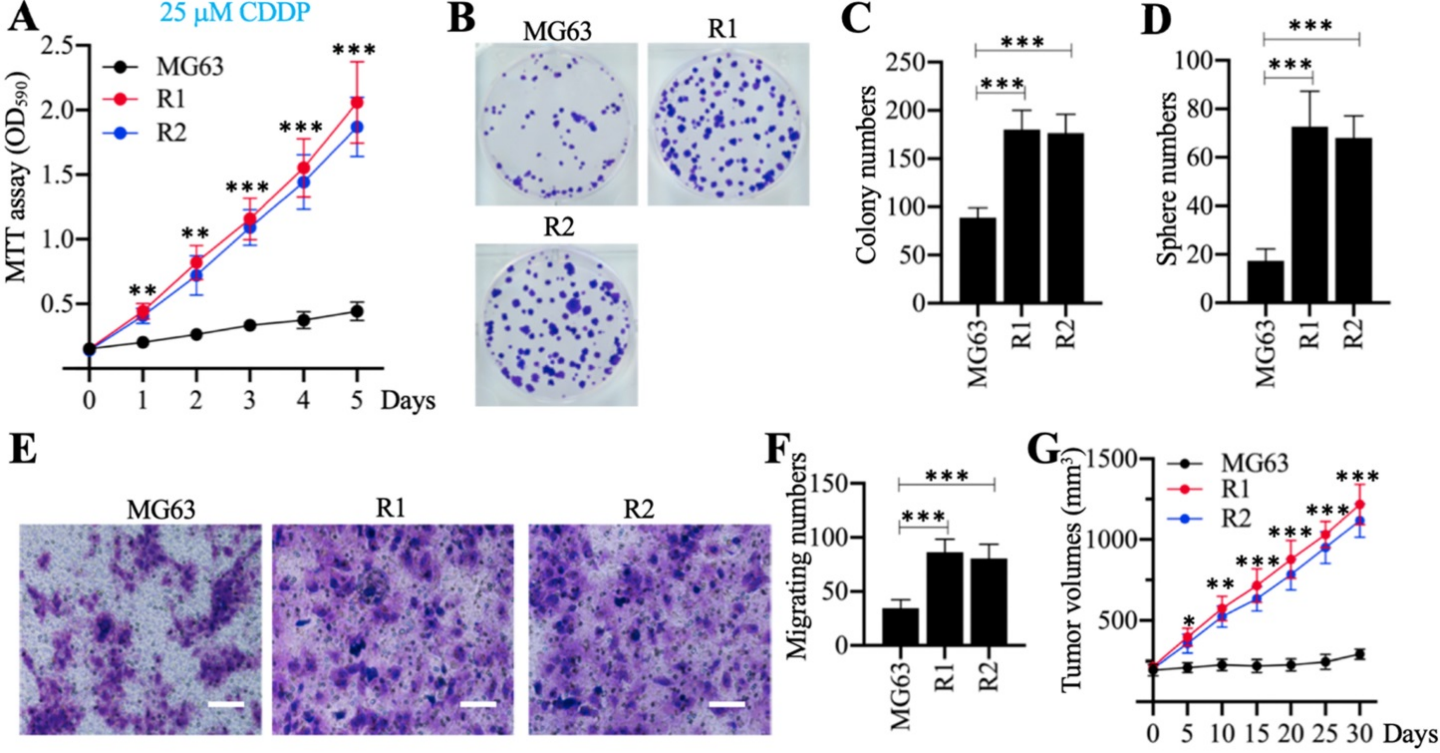
** The *in vitro* and *in vivo* phenotypes of MG63-R1/R2 cells following cisplatin (CDDP) treatment.** (A) MTT assay results. The same numbers of MG63, MG63-R1 and MG63-R2 cells were seeded into DMEM containing 25 µM cisplatin (CDDP) and cell viability was determined every day for five days. ** *P* < 0.01 and *** *P* < 0.001. (B and C) Colony formation results. The same numbers of MG63, MG63-R1, and MG63-R2 cells (approximately 500 cells) were seeded into six-well plates and grown in DMEM containing 25 µM CDDP. Colonies were stained with 0.1% crystal violet (B). Colony numbers were counted manually (C). ^***^P <0.001. (D) Sphere numbers. The same numbers of MG63, MG63-R1, and MG63-R2 cells were grown in sphere formation medium containing 25 µM CDDP. Sphere numbers were counted manually.^ ***^P <0.001. (E and F) Cell migration results. Cell suspensions in serum-free DMEM containing 25 µM CDDP were placed in Boyden chambers to determine cell migration. The migrated cells were stained with 0.1% crystal violet (E). Bars=50 µm. The numbers of crystal violet positive cells were counted manually (F). ^***^P <0.001. (G) Tumor volumes *in vivo*. The same volumes of MG63, MG63-R1, and MG63-R2 cell suspensions were injected into nude mice (n=10 for each cell line). Mice with similar tumor volumes (approximately 200 mm^3^) were injected with CDDP at 5-day intervals. Tumor volumes were determined at 5-day intervals for 30 days. ^*^P <0.05, ** *P* < 0.01 and ^***^P <0.001.

Cell phenotypes, including cell proliferation, colony formation, sphere formation, cell migration, and tumor growth *in vivo*, were evaluated in response to treatment with 25 µM CDDP. The MTT assays revealed that MG63-R1 and MG63-R2 cells had higher cell viability than the MG63 maternal cells after CDDP treatment (Figure 2A). Both cell lines showed similar phenotype patterns in terms of numbers of colonies, spheres, and migrating cells (Figures 2B-2F). MG63, MG63-R1, and MG63-R2 cell suspensions were then injected into nude mice generated tumors. Mice harboring tumors of similar volume (approximately 200 mm^3^) were administered CDDP at 5-days intervals. The tumor volumes were much larger in mice harboring MG63-R1 and MG63-R2 cells than in mice injected with MG63 cells (Figure 2G). The MG63-R1 and MG63-R2 cells were insensitive to CDDP, and their CSC properties could be used to study the mechanism of CSC-mediated chemoresistance.

### Both *CtBP1* and *MDR1* were overexpressed in MG63-R1/R2 cells and chemoresistant biopsies

**Figure 3:**
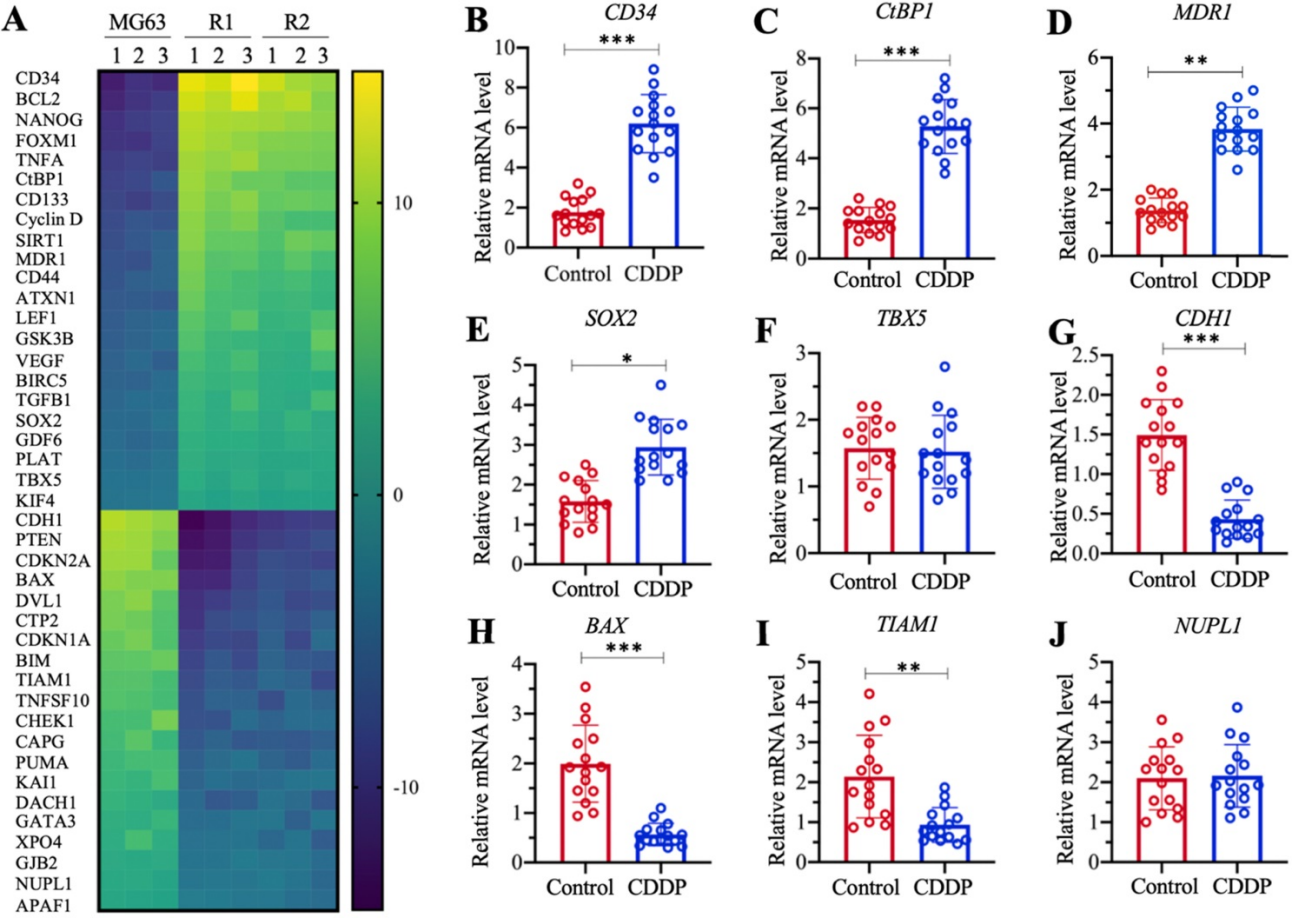
** Identification of the differentially expressed genes in MG63-R1/R2 cells and detection of nine representative genes in cisplatin (CDDP)-resistant biopsies.** (A) Microarray results. Three independent RNA samples from MG63, MG63-R1, and MG63-R2 cells were subjected to microarray analysis. (B-J) Detection of nine representative genes in CDDP-resistant biopsies. *CD34* (B),* CtBP1* (C), *MDR1* (D), *SOX2* (E), *TBX5* (F),* CDH1* (G), *BAX* (H), *TIAM1* (I), and *NUPL1* (J), were selected for expression determinations using 15 paired RNA samples from osteosarcoma patients without chemoresistance (Control) and with CDDP resistance.* P* <0.05, ** *P* < 0.01 and ^***^P <0.001.

The profiles of differentially expressed genes in MG63-R1 and MG63-R2 cells were examined by microarray analyses using three replicate RNA samples. We obtained a total of 42 genes whose expression levels were consistently decreased or increased in MG63-R1 and MG63-R2 cells compared to the MG63 maternal cells (Figure 3A and Supplementary Table 3). Among these aberrantly expressed genes, we found that *MDR1* was significantly induced in MG63-R1 and MG63-R2 cells (approximately 4.3-fold in R1 and 3.9-fold in MG63-R2). We also found significant upregulation of *CtBP1*, an activator of *MDR1*, in MG63-R1 and MG63-R2 cells (Figure 3A). Several proapoptotic genes, including *BAX*, *BIM*, *PUMA,* and *APAF1*** (**apoptotic protease activating factor 1), were downregulated, while two anti-apoptotic genes, *BIRC5* (baculoviral IAP repeat containing 5) and *BCL2*, were induced in MG63-R1 and MG63-R2 cells (Figure 3A). Several tumor suppressor genes, including *CDH1*, *PTEN*, *CDKN1A*, and *CDKN2A*, were downregulated in MG63-R1 and MG63-R2 cells (Figure 3A). Therefore, at least three classes of genes, including apoptotic genes, tumor suppressors, and *MDR1*, were differentially expressed in the MG63-R1 and MG63-R2 cells.

We also examined the aberrantly expressed genes identified in the microarray analyses in chemoresistant biopsies by collecting 15-paired biopsies from osteosarcoma patients without chemoresistance (Control) and with CDDP resistance, and then detected the expression of selected 9 randomly selected genes in the biopsies. These 9 genes included five upregulated genes (*CD34* [cluster of differentiation 34], *CtBP1*, *MDR1*, *SOX2* [SRY-related HMG-box gene 2], and *TBX5* [T-box protein 5]) and four downregulated genes (*CDH1*, *BAX*, *TIAM1* [T cell lymphoma invasion and metastasis 1], and *NUPL1* [nucleoporin-like protein 1]). The RT-qPCR analysis results showed increased expression of *CD34*, *CtBP1*, *MDR1,* and *SOX2* by approximately 3.8-fold, 3.5-fold, 3.2-fold, and 1.8-fold, respectively, in CDDP-resistant biopsies compared to nonresistant biopsies (Figures 3B-3E). By contrast,* TBX5* expression was not significantly changed (Figure 3F). The expression levels of *CDH1*, *BAX*, and *TIAM1* were decreased approximately 3.5-fold, 3.3-fold, and 2.5-fold, respectively, in CDDP-resistant biopsies compared to nonresistant biopsies (Figures 3G-3I). The expression of *NUPL1* was not changed in the two biopsy types (Figure 3J). The expression of some genes in the biopsies were not consistent with their expression levels in microarray results, but our results overall supported overexpression of both *CtBP1* and *MDR1* in MG63-R1 and MG63-R2 cells and in chemoresistant biopsies.

### CtBP1 assembled a complex with FOXM1 *in vitro* and *in vivo*

**Figure 4:**
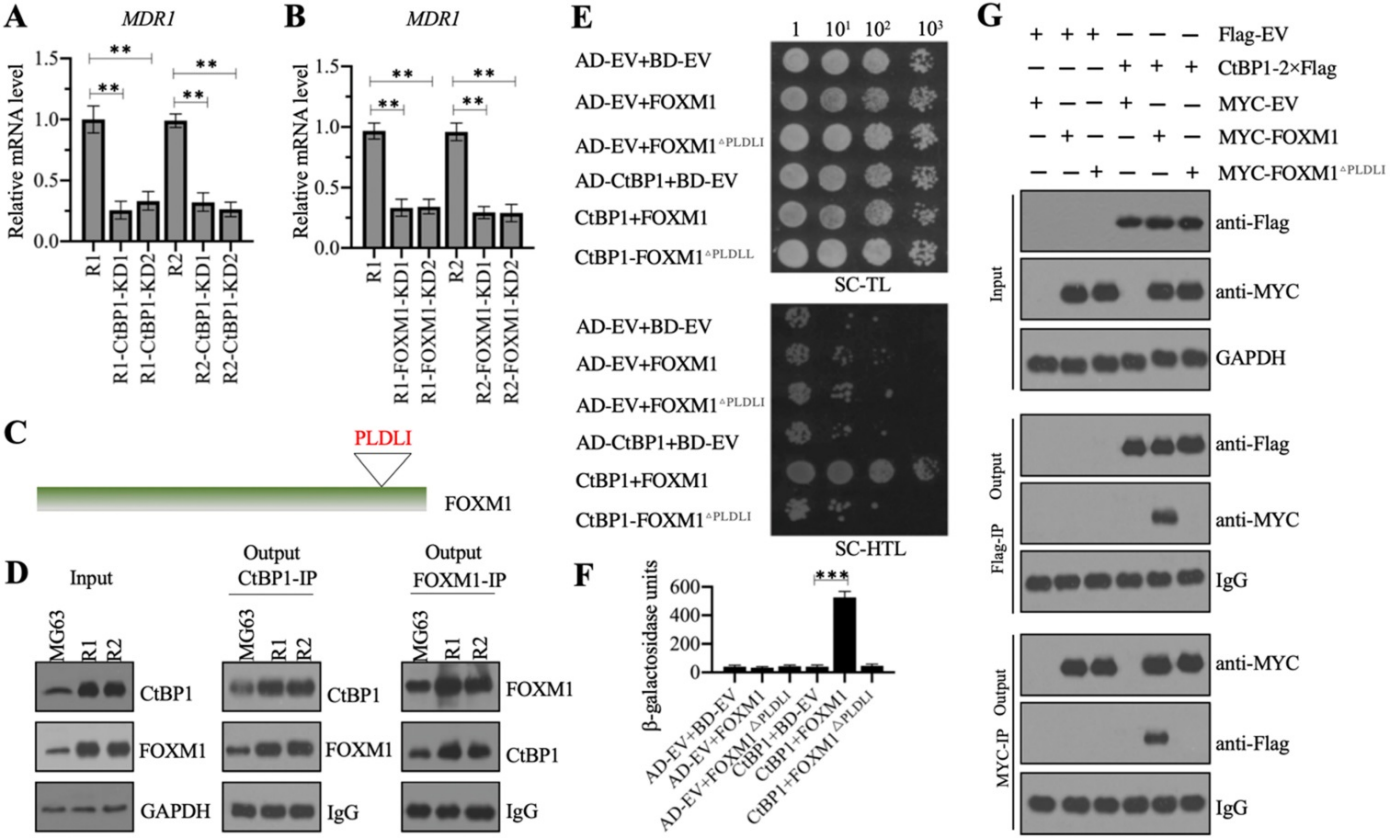
** CtBP1 directly interacted with FOXM1 *in vivo* and *in vitro.***(A and B) Knockdown of *CtBP1* or *FOXM1* significantly decreased the expression of *MDR1*. Total RNA samples from MG63-R1, MG63-R2, and two independent knockdown cell lines of CtBP1-KD (A), and two independent knockdown cell lines of FOXM1-KD (B) underwent RT-qPCR analyses to examine *MDR1* expression. ** *P* < 0.01. (C) The FOXM1 protein contained a PLDLI motif. (D) CtBP1 directly interacted with FOXM1 *in vivo*. Total cell lysates from MG63, MG63-R1, and MG63-R2 cells were subjected to IP assays using anti-CtBP1-coupled and anti-FOXM1-coupled Protein G beads. The input and output proteins were subjected to western blotting to detect CtBP1 and FOXM1 protein levels. GAPDH and IgG were the loading controls for input and output proteins, respectively. (E) CtBP1 directly interacted with FOXM1 in yeast cells. Yeast cells expressing different plasmids were subjected to plate-dotting assays on SC-TL and SC-HTL media with different dilution folds (1, 10^1^, 10^2^, and 10^3^). (F) β-galactosidase activity. Yeast cells used in (E) were applied to measure β-galactosidase activity.^ ***^P <0.001. (G) CtBP1 directly interacted with FOXM1 *in vitro*. MG63 cells expressing different plasmids were subjected to co-IP assays using Flag- and Myc-agarose beads. The input and output proteins were subjected to western blotting to detect CtBP1 and FOXM1 protein levels. GAPDH and IgG were the loading controls of input and output proteins, respectively.

We investigated whether CtBP1 could activate the expression of *MDR1* in MG63-R1 and MG63-R2 cells, as it does in NCI/ADR-RES and A2780/DX cells, by generating two independent CtBP1 knockdown cell lines in the MG63-R1 and MG63-R2 backgrounds. The mRNA and protein levels of CtBP1 were determined in these cells to verify its successful suppression (Supplementary Figures 1A-1C). The same RNA samples were also used to detect the *MDR1* mRNA level. The RT-qPCR results showed that knockdown of *CtBP1* decreased the *MDR1* mRNA level (Figure 4A), suggesting that *MDR1* expression was dependent on *CtBP1*. CtBP1 is a transcriptional regulator, rather than a transcription factor, so it does not directly bind to its target gene promoters to initiate transcription. Analysis of the microarray results (Figure 3A) revealed upregulation of the FOXM1 transcription factor in MG63-R1 and MG63-R2 cells.

We also detected *FOXM1* mRNA expression in 15-paired CDDP-resistant biopsies and nonresistant biopsies. As shown in Supplementary Figure 1D, we observed a significant induction of *FOXM1* mRNA level (approximately 3.1-fold) in CDDP-resistant biopsies compared to nonresistant biopsies. We also generated two independent FOXM1 knockdown cell lines in MG63-R1 and MG63-R2 background, respectively (Supplementary Figures 1 E-1G). Using RNA samples isolated from these cells, we also found decreased *MDR1* mRNA levels following knockdown of *FOXM1* (Figure 4B).

The similar expression patterns of *CtBP1* and *FOXM1* in MG63-R1 and MG63-R2 cells and in CDDP-resistant biopsies prompted us to investigate a possible direct interaction between the two genes. CtBP1 conservatively interacts with other proteins through its PXDLX motif, so and we identified a PLDLI motif in the C-terminal of FOXM1 (Figure 4C and Supplementary Figure 2), suggesting a high possibility for direct interaction between CtBP1 and FOXM1. We verified this possibility by IP experiments using both anti-CtBP1 and anti-FOXM1 antibodies in lysates of MG63, MG63-R1, and MG63-R2 cells. CtBP1 and FOXM1 both pulled down each other (Figure 4C). We also co-transformed yeast cells with pGADT7+pGBKT7, pGADT7+pGBKT7-FOXM1, pGADT7+pGBKT7-FOXM1^△PLDLI^, pGADT7-CtBP1+pGBKT7, pGADT7-CtBP1+pGBKT7-FOXM1, or pGADT7-CtBP1+pGBKT7-FOXM1^△PLDLI^ and examined their growth in SC-TL and SC-HTL media. Only cells co-expressing CtBP1 and FOXM1, but not CtBP1 and FOXM1^△PLDLI^, grew in SC-HTL medium (Figure 4E). Only cells co-expressing CtBP1 and FOXM1, but not CtBP1 and FOXM1^△PLDLI^, showed increases in β-galactosidase activity (Figure 4F). Co-IP assays to confirm the direct interaction of CtBP1 and FOXM1 also showed that only the wild-type FOXM1, but not FOXM1^△PLDLI^, could directly interact with CtBP1 (Figure 4G). Taken together, these results indicated a direct interaction between CtBP1 and FOXM1 through the PLDLI motif.

### Knockdown of either *CtBP1* or *FOXM1* significantly increased the chemosensitivity of MG63-R1 and MG63-R2 cells

**Figure 5:**
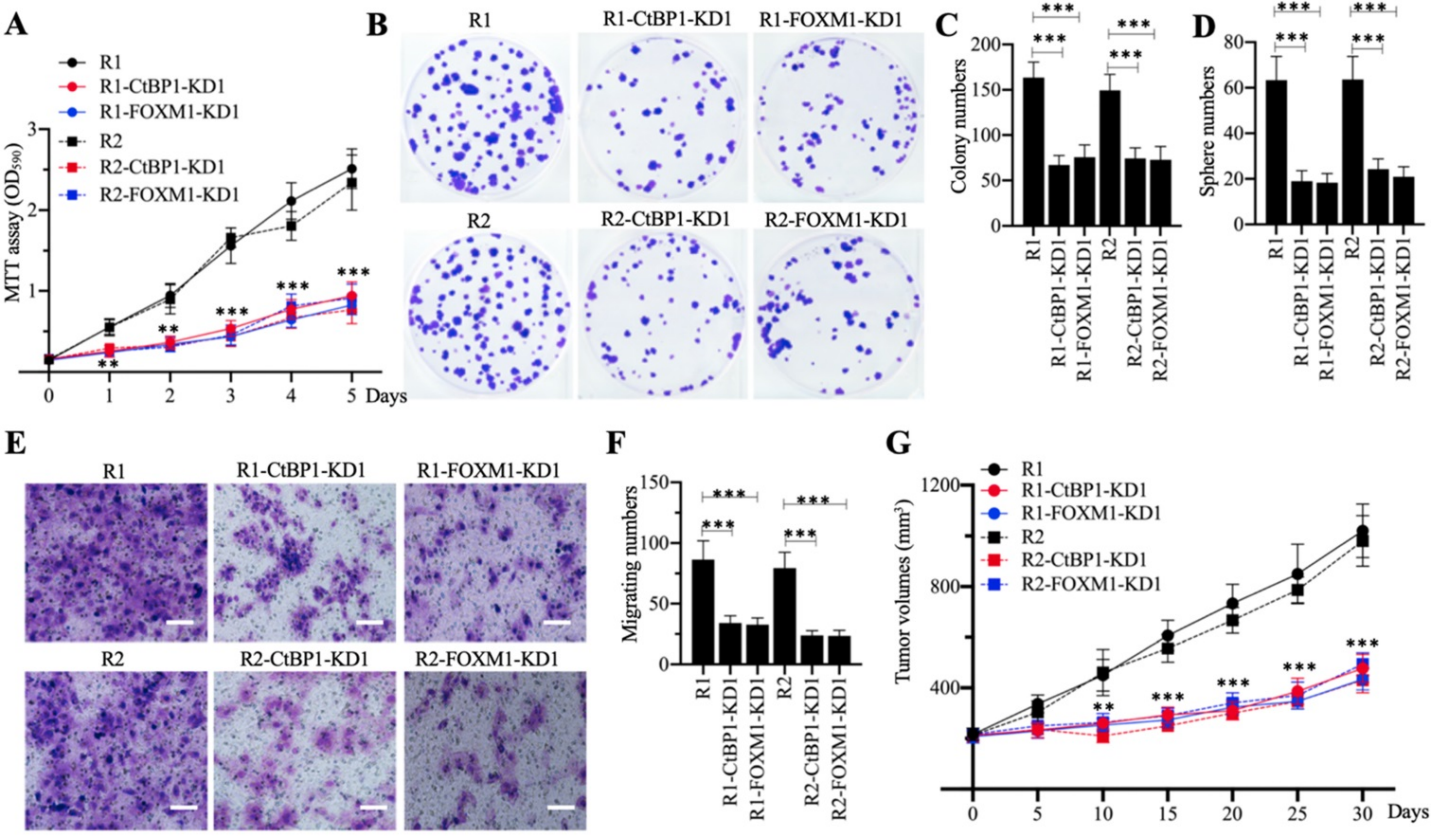
** The *in vitro* and *in vivo* phenotypes of CtBP1-KD and FOXM1-KD cells under CDDP treatment.** (A) MTT assay results. The same numbers of MG63-R1, MG63-R1-CtBP1-KD1, MG63-R1-FOXM1-KD1, MG63-R2, MG63-R2-CtBP1-KD1, and MG63-R2-FOXM1-KD1 cells were seeded into DMEM containing 25 µM CDDP and cell viability was determined every day for five days. ** *P* < 0.01 and *** *P* < 0.001. (B and C) Colony formation results. The same numbers of cells (approximately 500) used in (A) were seeded into six-well plates and grown in DMEM containing 25 µM CDDP. Colonies were stained with 0.1% crystal violet (B). Colony numbers were counted manually (C). ^***^P <0.001. (D) Sphere numbers. The same numbers of cells used in (A) were grown in sphere formation medium containing 25 µM CDDP. Sphere numbers were counted manually.^ ***^P <0.001. (E and F) Cell migration results. Cell suspensions in serum-free DMEM containing 25 µM CDDP were placed in Boyden chambers to determine cell migration. The migrated cells were stained with 0.1% crystal violet (E). Bars=50 µm. The numbers of crystal violet positive cells were counted manually (F). ^***^P <0.001. (G) Tumor volumes *in vivo*. The same volumes of cell suspension as indicated in the figure were injected into nude mice (n=10 for each cell line). Mice harboring similar tumor volumes (approximately 200 mm^3^) were injected with CDDP at 5-day intervals. Tumor volumes were determined at 5-day intervals for 30 days. ** *P* < 0.01 and ^***^P <0.001.

Knockdown of *CtBP1* or *FOXM1* in MG63-R1 and MG63-R2 cells decreased the expression of *MDR1*, suggesting that CtBP1-KD and FOXM1-KD cells would show increased chemosensitivity. Cell proliferation studies confirmed a significant decrease in viability in CtBP1-KD and FOXM1-KD cells in comparison to MG63-R1 and MG63-R2 cells after CDDP treatment (Figure 5A). Similarly, the numbers of colonies, spheres, and migrating cells were also markedly decreased in CtBP1-KD and FOXM1-KD cells following CDDP treatment (Figures 5B-5F). The administration of CDDP also significantly decreased the tumor volumes in mice injected with CtBP1-KD or FOXM1-KD cells when compared with mice injected with MG63-R1 orMG63-R2 cells (Figure 5G). The knockdown of either *CtBP1* or *FOXM1* therefore appeared to significantly increase the chemosensitivity of MG63-R1 and MG63-R2 cells both *in vitro* and *in vivo*.

### The CtBP1-FOXM1 complex bound to the promoter of *MDR1* to activate its expression

**Figure 6:**
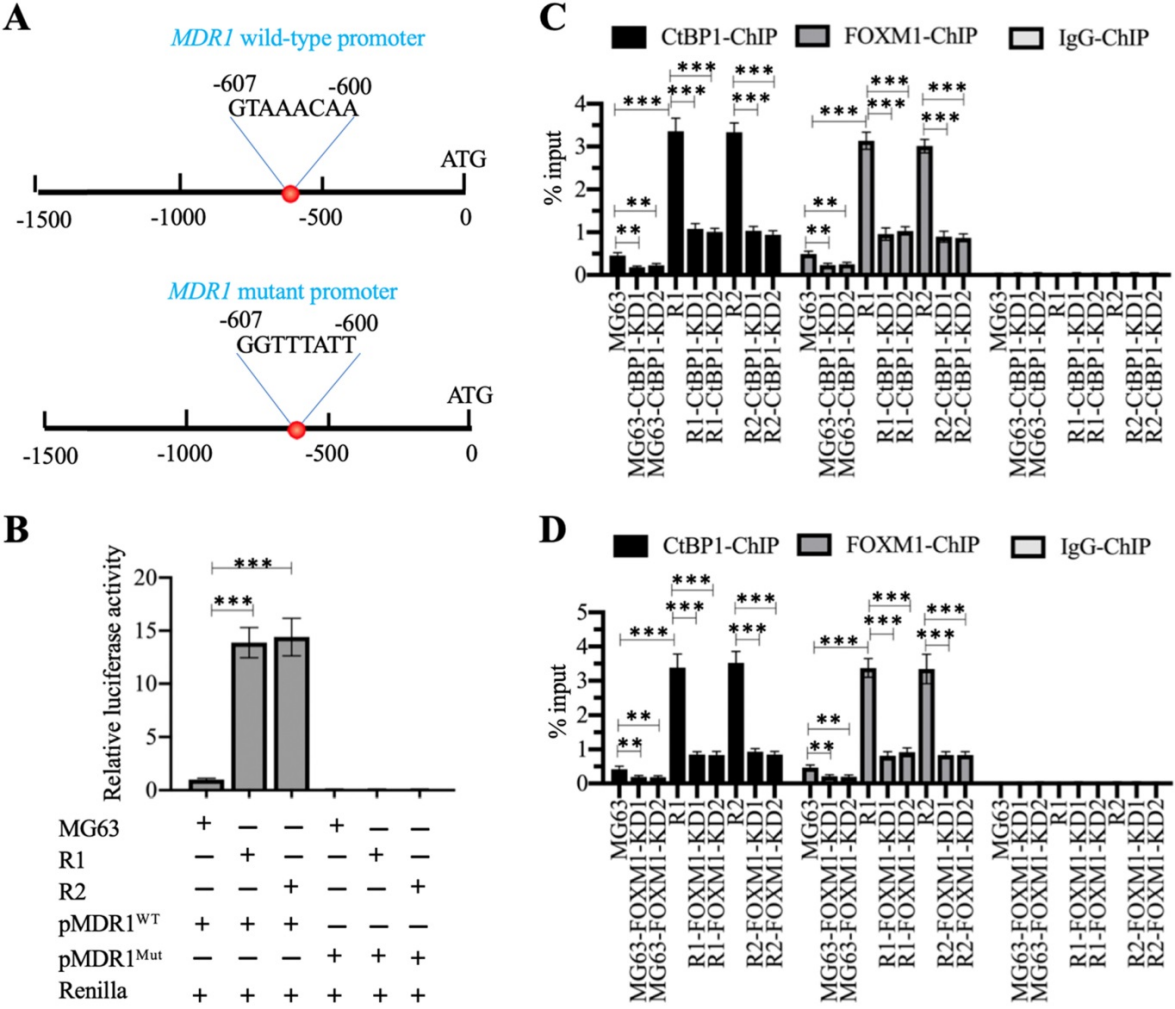
** The CtBP1-FOXM1 complex transactivated the expression of *MDR1* by binding to the *MDR1* promoter.** (A) Schematic diagrams of *MDR1* WT and mutant promoters. (B) Luciferase activity. ^***^P <0.001. (C) ChIP results in CtBP1-KD cells. The MG63, MG63-CtBP1-KD1/2, MG63-R1, MG63-R1-CtBP1-KD1/2, MG63-R2, and MG63-R2-CtBP1-KD1/2 cells were used for ChIP assays with anti-CtBP1, anti-FOXM1, and IgG. The purified DNA was subjected to RT-qPCR analyses to determine the occupancies of CtBP1 and FOXM1 on the *MDR1* promoter. ** *P* < 0.01 and ^***^P <0.001. (D) ChIP results in FOXM1-KD cells. The MG63, MG63-FOXM1-KD1/2, MG63-R1, MG63-R1-FOXM1-KD1/2, MG63-R2, and MG63-R2-FOXM1-KD1/2 cells were used for ChIP assays with anti-CtBP1, anti-FOXM1, and IgG. The purified DNA was subjected to RT-qPCR analyses to determine the occupancies of CtBP1 and FOXM1 on the *MDR1* promoter. ** *P* < 0.01 and ^***^P <0.001.

We also analyzed the promoter of *MDR1* (1500-bp length) to determine the presence of the FOXM1 binding site using its consensus sequence (5′-G(C/T)AAA(T/C)AA-3′). We identified a FOXM1 binding site (5′-GTAAACAA-3′) localized between nucleotides -600 and -607 on the *MDR1* promoter (Figure 6A). Firefly luciferase vectors containing the wildtype (WT) or mutated promoter of *MDR1* (Figure 6A) confirmed much higher luciferase activities in MG63-R1 and MG63-R2 cells co-transfected with pGL4.26-pMDR1^WT^ + Renilla than in MG63 cells (Figure 6B). However, the luciferase activities were similar in MG63-R1 and MG63-R2 cells co-transfected pGL4.26-pMDR1^Mut^ + Renilla and in MG63 cells and all activities were much lower than the activity observed in MG63 cells co-transfected with pGL4.26-pMDR1^WT^ + Renilla (Figure 6C). The FOXM1 binding site was therefore required for the induction of *MDR1* expression.

We also performed ChIP assays on CtBP1-KD and FOXM1-KD cells in MG63, MG63-R1, and MG63-R2 backgrounds using anti-CtBP1, anti-FOXM1, and IgG to determine the enrichment of the CtBP1-FOXM1 complex on the *MDR1* promoter. The occupancies of CtBP1 and FOXM1 showed similar patterns (Figure 6C and 6D), and showed significant decreases when CtBP1 and FOXM1 were knocked down in the three backgrounds (Figures 6C and 6D). Comparison of the occupancies in MG63-R1 and MG63-R2 cells with MG63 cells revealed that both CtBP1 and FOXM1 were significantly enriched on the *MDR1* promoter (Figures 6C and 6D), providing evidence that CtBP1 and FOXM1 could dock onto the *MDR1* promoter to activate *MDR1* expression.

### Blocking the CtBP1-FOXM1 complex with specific inhibitors significantly decreased *MDR1* expression

**Figure 7:**
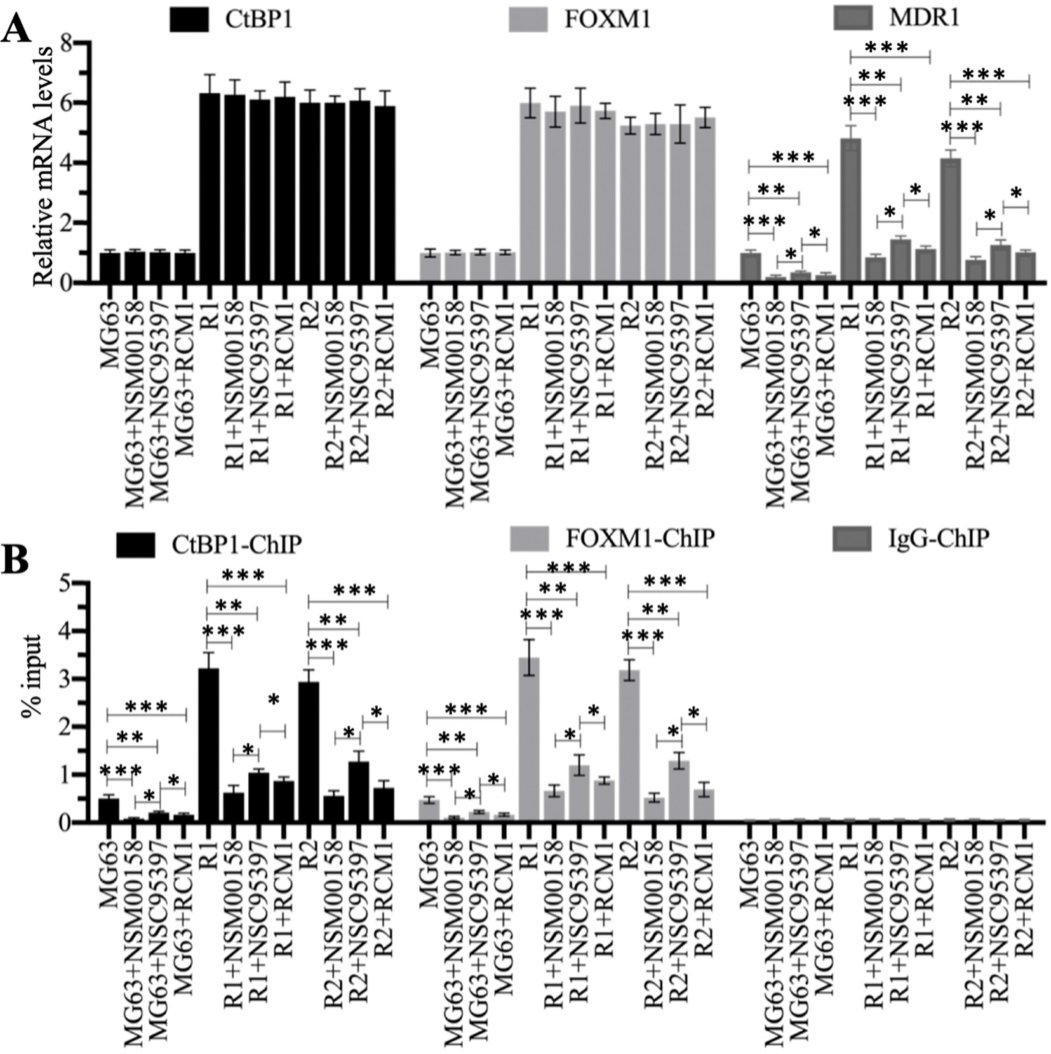
** Blockage of the CtBP1-FOXM1 complex with small molecules significantly decreased the expression of *MDR1.***(A) The relative mRNA levels of *CtBP1*, *FOXM1,* and *MDR1*. Total RNA samples were subjected to RT-qPCR analyses to examine the mRNA expression levels for *CtBP1*, *FOXM1,* and *MDR1*. * *P* < 0.05, ** *P* < 0.01 and *** *P* < 0.001. (B) ChIP results. ChIP assays were performed using anti-CtBP1, anti-FOXM1, and IgG, respectively. The purified DNA was subjected to RT-qPCR analyses to determine the occupancies of CtBP1 and FOXM1 on the promoter of *MDR1*. * *P* < 0.05, ** *P* < 0.01 and ^***^P <0.001.

We treated MG63, MG63-R1, and MG63-R2 cells with two CtBP1 inhibitors (NSM00158 and NSC95397) and one FOXM1 inhibitor (RCM1) (see Supplementary Figure 3 for the chemical structures of these three small molecules). The reported IC_50_ values of these compounds were approximately 2 µM for NSM00158 20, approximately 20 µM for NSC95397 24, and approximately 1 µM for RCM1 25, so we used these IC_50_ concentrations to treat the cells. The RT-qPCR results for the treated cells showed no changes in the mRNA levels of *CtBP1* and *FOXM1* (Figure 7A), but varying degrees of suppression of *MDR1* expression (Figure 7A). Cells treated with NSM00158 and RCM1 showed only one fifth of the *MDR1* expression seen in untreated cells and cells treated with NSC95397 showed only one fourth of the *MDR1* expression (Figure 7A). The CtBP1, FOXM1, and MDR1 protein levels were consistent with their corresponding mRNA levels (Supplementary Figures 4A and 4B). ChIP assays with anti-CtBP1, anti-FOXM1, and IgG, conducted to examine the enrichment of CtBP1-FOXM1 complex on the *MDR1* promoter, revealed a reduction in the occupancies of CtBP1 and FOXM1 in NSM00158-treated or RCM1-treated cells to one-fifth of the occupancy observed in untreated cells, while cells treated with NSC95397 showed a decrease to one-fourth the occupancy observed in untreated cells (Figure 7B). These inhibitors of the CtBP1-FOXM1 complex therefore decreased the enrichment of CtBP1 and FOXM1 on the *MDR1* promoter, thereby repressing *MDR1* expression. The inhibition of *MDR1* expression was much stronger with NSM00158 and RCM1 than with NSC95937, suggesting that NSM00158 and RCM1 might be effective drug candidates for overcoming chemoresistance through repression of *MDR1* expression.

### NSM00158, NSC95397, and RCM1 significantly inhibited MG63-R1 and MG63-R2 cell growth *in vitro* and tumor growth *in vivo*

**Figure 8:**
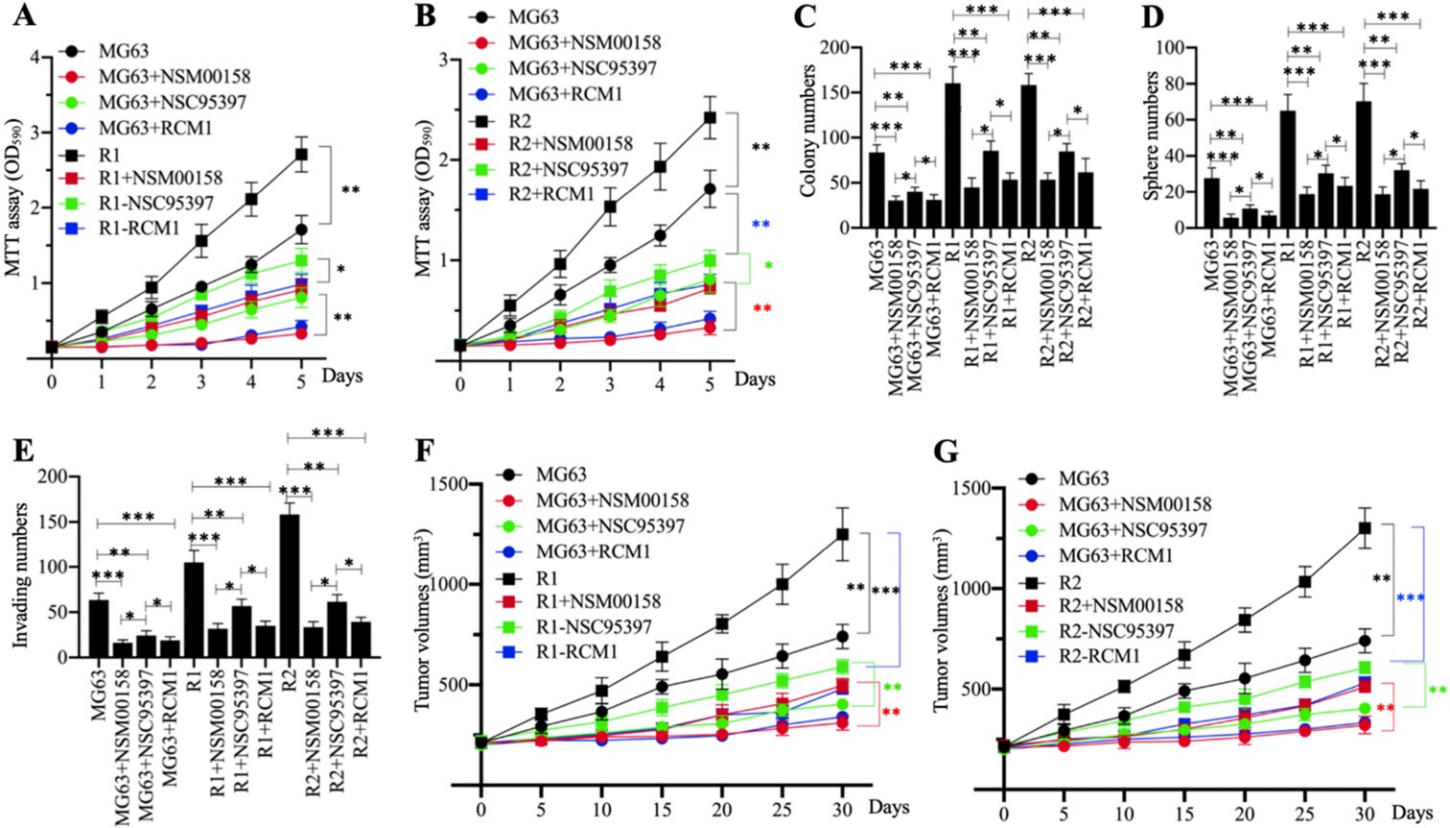
** The *in vitro* and *in vivo* phenotypes of small molecule-treated cells.** (A and B) MTT assay results. The same numbers of MG63, MG63-R1 (A), and MG63-R2 (B) cells were seeded into DMEM containing 2 µM NSM00158, 20 µM NSC95397, or 1 µM RCM1 and cell viability was determined every day for five days. * *P* < 0.05 and ** *P* < 0.01. (C) Colony numbers. The same numbers of MG63, MG63-R1, and MG63-R2 cells (approximately 500) were seeded into six-well plates and grown in DMEM containing 2 µM NSM00158, 20 µM NSC95397, or 1 µM RCM1. Colony numbers were counted manually. * *P* < 0.05, ** *P* < 0.01, ^***^P <0.001. (D) Sphere numbers. The same numbers of MG63, MG63-R1, and MG63-R2 cells were grown in sphere formation medium containing 2 µM NSM00158, 20 µM NSC95397, or 1 µM RCM1. Sphere numbers were counted manually.^ ***^P <0.001. (E) Migrating cell numbers. Cell suspensions in serum-free DMEM containing 2 µM NSM00158, 20 µM NSC95397, or 1 µM RCM1 were placed in Boyden chambers to determine cell migration. The numbers of crystal violet positive cells were counted manually. * *P* < 0.05, ** *P* < 0.01, ^***^P <0.001. (F and G) Tumor volumes *in vivo*. The same volumes of cell suspension as indicated in the figure were injected into nude mice (n=10 for each cell line). Mice harboring similar tumor volumes (approximately 200 mm^3^) were injected with NSM00158, NSC95397, or RCM1 at 5-day intervals. Tumor volumes were determined at 5-day intervals for 30 days. ** *P* < 0.01 and ^***^P <0.001.

Cell proliferation in MG63, MG63-R1, and MG63-R2 cells treated with 2 µM NSM00158, 20 µM NSC95397, and 1 µM RCM1 revealed significant reductions in cell proliferation. NSM00158 and RCM1 showed a similar inhibitory effect in all three cell backgrounds and the effects were stronger than those of NSC95397 (Figures 8A and 8B). The similar inhibitory patterns were observed for numbers of colonies, spheres, and migrating cells following treatment with the three small molecules (Figures 8C-8E and Supplementary Figure 5). Tumors (approximately 200 mm^3^ in size) in nude mice, induced by injection of MG63, MG63-R1, and MG63-R2 cells, responded to *in vivo* administration of NSM00158, NSC95937, and RCM1 at 5-day intervals by significant decreases in growth (Figures 8F and 8G). Consistent with the *in vitro* data, the effect was stronger with NSM00158 and RCM1 than with NSC95397 (Figures 8F and 8G).

### The combination of NSM00158 and CDDP significantly reversed chemoresistance

**Figure 9:**
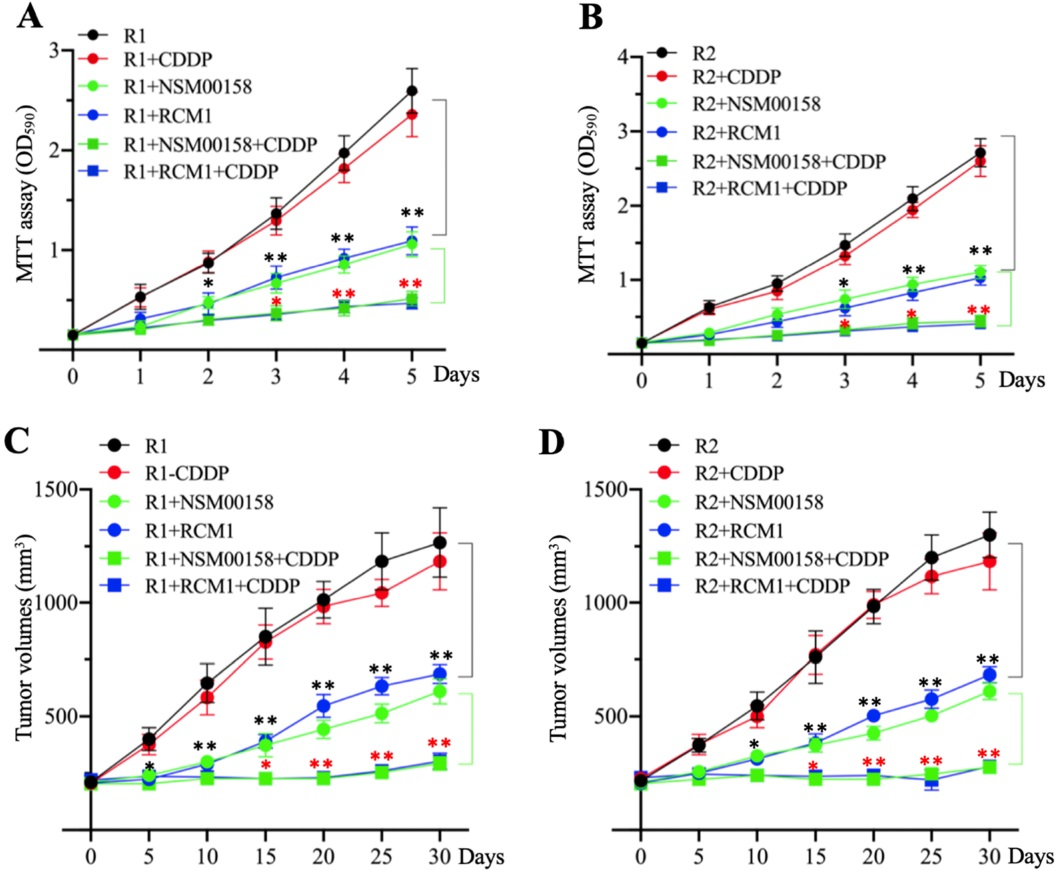
** The *in vitro* and *in vivo* effects of small molecules in combination with cisplatin (CDDP) on MG63-R1 and MG63-R2 cell growth.** (A and B) MTT assay results. The same numbers of MG63-R1 (A) and MG63-R2 (B) cells were seeded into DMEM containing 25 µM CDDP, 2 µM NSM00158, 1 µM RCM1, 2 µM NSM00158+25 µM CDDP, or 1 µM RCM1+25 µM CDDP and cell viability was determined every day for five days. * *P* < 0.05 and ** *P* < 0.01. (C and D) Tumor volumes *in vivo*. The same volumes of cell suspension of MG63-R1 (C) and MG63-R2 (D) were injected into nude mice (n=10 for each cell line). Mice harboring similar tumor volumes (approximately 200 mm^3^) were further injected with CDDP, NSM00158, RCM1, NSM00158+CDDP, or RCM1+CDDP at 5-day intervals. Tumor volumes were determined at 5-day intervals for 30 days. ** *P* < 0.01 and ^***^P <0.001.

The similar inhibitory effects of NSM00158 and RCM1 on MG63-R1 and MG63-R2 cell growth *in vitro* and *in vivo* prompted us to examine the potential synergistic effects these two small molecules in combination with CDDP. Due to the similar phenotype patterns of cell proliferation, colony formation, sphere formation, and cell migration in MG63, MG63-R1, and MG63-R2 cells in response to NSM00158 or RCM1 treatments, we only determined cell proliferation in MG63-R1 and MG63-R2 cells as a representative experiment and we treated the cells with 2 µM NSM00158, 1 µM RCM1, 25 µM CDDP, 2 µM NSM00158+25 µM CDDP, or 1 µM RCM1+25 µM CDDP. The treatment with NSM0018+CDDP or RCM1+CDDP reduced the viability of both cell types by approximately 80% when compared to untreated or CDDP-treated cells. Tumors (approximately 200 mm^3^ in size) formed by injection of MG63-R1 and MG63-R2 cells into nude mice responded to combined NSM00158+CDDP or RCM1+CDDP treatments by an 85-90% reduction in tumor volumes compared to untreated or CDDP-treated mice (Figures 9C and 9D). These results confirmed NSM00158 and RCM1 as two promising candidates for overcoming chemoresistance in osteosarcomas.

## Discussion

**Figure 10:**
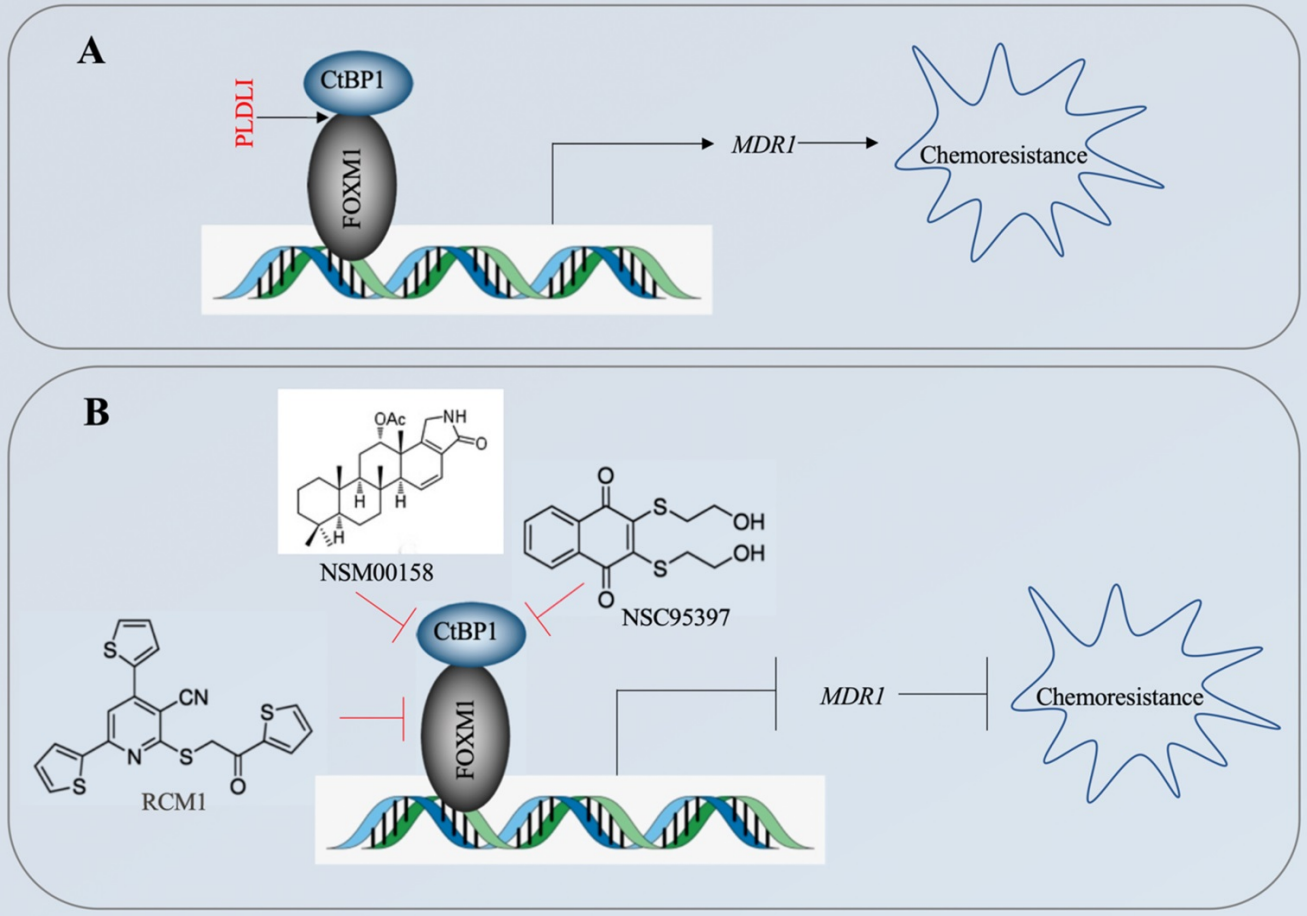
** A schematic diagram for targeting the CtBP1-FOXM1 complex with small molecules to decrease *MDR1* expression in osteosarcoma CSCs.** (A) A schematic diagram of the transactivation of *MDR1* by the CtBP1-FOXM1 complex. FOXM1 directly interacts with CtBP1, and this complex specifically binds to the *MDR1* promoter to transactivate *MDR1* expression. The overexpressed MDR1 effluxes the chemotherapeutic drug, causing chemoresistance. (B) A schematic diagram of targeting the CtBP1-FOXM1 complex with small molecules to decrease *MDR1* expression in osteosarcoma CSCs. Two CtBP1 inhibitors (NSM00158 and NSC95397) and one FOXM1 inhibitor (RCM1) can disrupt the binding of the CtBP1-FOXM1 complex to the *MDR1* promoter, thereby inhibiting the expression of *MDR1* and increasing chemosensitivity.

The CtBP1-mediated transactivation of *MDR1* in multidrug-resistant breast cancer cells has been known for more than ten years 15. However, the CtBP1-coupled transcription factor involved in this transactivation process has not been identified. In the present study, we revealed that CtBP1 directly interacted with FOXM1, and that the complex docked onto the *MDR1* promoter through a specific binding site (5′-GTAAACAA-3′) to activate *MDR1* expression in osteosarcoma CSCs. The overexpressed MDR1 served as a transporter that promoted the efflux of chemotherapeutic drugs, causing chemoresistance (Figure 10A). Importantly, the knockdown of either *CtBP1* or *FOXM1* and targeting of the CtBP1-FOXM1 complex members with specific inhibitors, including NSM0018 and NSC95397 for CtBP1, and RCM1 for FOXM1, significantly decreased the MDR1 level and increased the chemosensitivity of osteosarcoma CSCs (Figure 10B).

Osteosarcoma CSCs, as a small class of cells capable of self-renewal and differentiation, are considered a major cause of cancer progression, metastasis, and chemoresistance 6. However, a detailed gene profile of differentially expressed genes is lacking for these CSCs. Our microarray analysis of CDDP-resistant osteosarcoma CSCs identified 42 differentially expressed genes whose expression was consistent in two different cell lines (MG63-R1 and MG63-R2) (Figure 3A and Supplementary Table 3). In addition to *MDR1* expression, we also found significant downregulation of several CtBP1-repressed targets, including *CDH1*, *BAX*, *BIM,* and *PTEN*. Among these genes, *CDH1* is regulated by the CtBP1-ZEB1/2 (zinc finger E-box binding homeobox 1/2) transcriptional complex 26, whereas *BAX* and *BIM* expression can be repressed by the CtBP1-p300-FOXO3a (forkhead box O3a) complex in osteosarcoma cells 27. FOXO3a is a homologous protein of FOXM1, but we did not find its overexpression or downregulation in MG63-R1 and MG63-R2 cells (Figure 3A and Supplementary Table 3). One possibility that could explain the downregulation of *BAX* and *BIM* is that they are controlled by other transcription factors. Another possible explanation is that CtBP1 also can assemble in a complex with p300 and FOXO3a to repress *BAX* and *BIM* expression. Currently, the nature of the CtBP1-coupled transcription factor that controls *PTEN* expression is not known.

The phenotypes of MG63-R1/R2-CtBP1-KD and MG63-R1/R2-FOXM1-KD cells, as well as the phenotypes of NSM00158-treated MG63-R1/R2 and RCM1-treated MG63-R1/R2 cells, revealed that knockdown or blockage of CtBP1 and FOXM1 caused similar effects on cell proliferation, colony formation, sphere formation, cell migration, and suppression of tumor growth. These similar responses suggest that CtBP1 may not participate in the aberrant expression of *CDH1*, *BAX*, *BIM,* and *PTEN* observed in osteosarcoma CSCs, as greater phenotype effects would have been expected following knockdown of *CtBP1* or NSM00158 treatment than following *FOXM1* knockdown or RCM1 treatment. Future studies will focus on the possible CtBP1 dependence of the downregulation of *CDH1*, *BAX*, *BIM,* and *PTEN* in osteosarcoma CSCs.

NSM00158 is a newly identified small molecule that disrupts CtBP2 function and impairs the functioning of the CtBP2-p300-Runx2 (Runt-related transcription factor 2) complex, thereby overcoming nonunion after bone fracture 20. The high homology of CtBP1 and CtBP2 led us to speculate that NSM00158 could also target CtBP1, and our results confirmed that NSM00158 targeted CtBP1 and inhibited the transactivation of *MDR1*, thereby increasing the chemosensitivity of osteosarcoma CSCs. This is a new function for NSM00158 and supports its development as a targeted drug for CtBP1/2 responses.

Several publications have reported that *MDR1* can be transactivated by three transcription factors: NF-κB, p53, and YBX1 28-30. Our microarray results revealed only FOXM1 as a differentially expressed transcription factor in osteosarcoma cells. We did not detect the expression of these three transcription factors in 15-paired clinical biopsies derived from chemoresistant osteosarcoma patients, but our *in vitro* and *in vivo* results obtained by knocking down or the blocking CtBP1-FOXM1 complex confirmed a significant improvement in chemoresistance, suggesting a dominant role for the CtBP1-FOXM1 complex at least in the regulation of *MDR1* expression. The promising *in vitro* results of for MG63-R1 and MG63-R2 cells and the *in vivo* tumor results observed with the combinations of NSM00158+CDDP and RCM1+CDDP suggest that targeting the CtBP1-FOXM1 complex prior to supplying chemotherapeutic drugs may significantly reduce osteosarcoma chemoresistance.

In summary, our findings support a role for specific binding of the CtBP1-FOXM1 transcriptional complex to the *MDR1* promoter to transactivate MDR1 expression in osteosarcoma CSCs and trigger chemoresistance. Targeting the CtBP1-FOXM1 complex members with specific small molecule inhibitors can significantly overcome this chemoresistance, suggesting a new therapeutic option for the treatment of osteosarcoma, especially in those patients with chemoresistance.

## Supplementary Material

Supplementary figures and tables.
