# Supplementary material for: Targeting the CtBP1-FOXM1 transcriptional complex with small molecules to overcome MDR1-mediated chemoresistance in osteosarcoma cancer stem cells
Source: J Cancer. 2021 Jan 1;12(2):482–97. doi: 10.7150/jca.50255 (PMC7739006; doi:10.7150/jca.50255)
Supplement: Supplementary file 1 — Supplementary figures and tables. [file jcav12p0482s1.pdf]

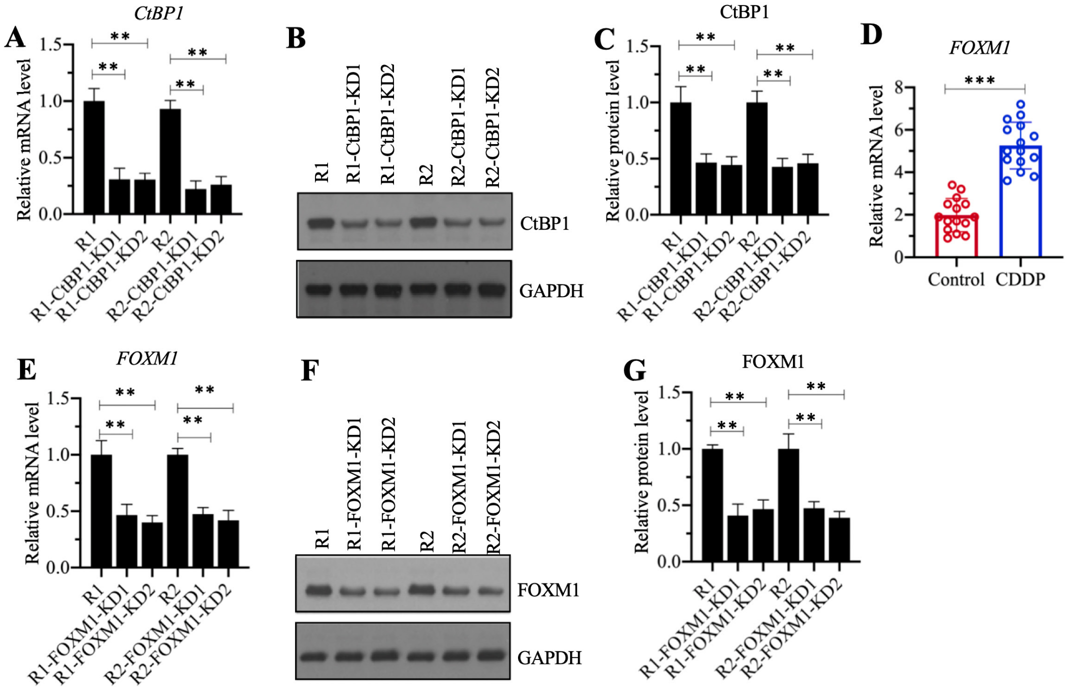

Supplementary Figure 1. Detection of CtBP1 or FOXM1 mRNA and protein levels in their corresponding knockdown cells

(A) The relative mRNA level of *CtBP1*. Total RNA samples isolated from the MG63-R1, MG63-R1-CtBP1-KD1, MG63-R1-CtBP1-KD2, MG63-R2, MG63-R2-CtBP1-KD1, and MG63-R2-CtBP1-KD2 cells were subjected to RT-qPCR analysis to examine *CtBP1* mRNA level. **(B** and **C)** The protein level of CtBP1. Cells used in (A) were subjected to western blotting to examine protein levels of CtBP1 and GAPDH (loading control) **(B)**. Protein signals in (B) were quantified and normalized to their corresponding GAPDH **(C)**. $^{**}P < 0.01$. **(D)** The *FXOM1* mRNA level in 15-paired biopsies. The same RNA samples used in Figure 3B were subjected to RT-qPCR analyses to examine *FXOM1* mRNA level. $^{***}P < 0.001$. **(E)** The relative mRNA level of *FOXM1*. Total RNA samples isolated from the MG63-R1, MG63-R1-FOXM1-KD1,

MG63-R1-FOXM1-KD2, MG63-R2, MG63-R2-FOXM1-KD1, and MG63-R2-FOXM1-KD2 cells were subjected to RT-qPCR analysis to examine *FOXM1* mRNA level. **(F** and **G)** The protein level of FOXM1. Cells used in (E) were subjected to western blotting to examine protein levels of FOXM1 and GAPDH (loading control) **(F)**. Protein signals in (F) were quantified and normalized to their corresponding GAPDH **(G)**. $^{**}P$ <0.01.

Human FOXM1 protein sequence

MKTSPRRPLILKRRRLPLPVQNAPSETSEEEPKRSPAQQESNQAEASKEVAESNSCKFPA
GIKIINHPTMPNTQVVAIPNNANIHSIITALTAKGKESGSSGPNKFILISCGGAPTQPPG
LRPQTQTSYDAKRTEVTLETLGPKPAARDVNLPRPPGALCEQKRETCADGEAAGCTINNS
LSNIQWLRKMSSDGLGSRSIKQEMEEKENCHLEQRQVKVEEPSRPSASWQNSVSERPPYS
YMAMIQFAINSTERKRMTLKDIYTWIEDHFPYFKHIAKPGWKNSIRHNLSLHDMFVRETS
ANGKVSFWTIHPSANRYLTLDQVFKPLDPGSPQLPEHLESQQKRPNPELRRNMTIKTELP
LGARRKMKPLLPRVSSYLVPIQFPVNQSLVLQPSVKVPLPLAASLMSSELARHSKRVRIA
PKVFGEQVVFGYMSKFFSGDLRDFGTPITSLFNFIFLCLSVLLAEEGIAPLSSAGPGKEE
KLLFGEGFSPLLPVQTIKEEEIQPGEEMPHLARPIKVESPPLEEWPSPAPSFKEESSHSW
EDSSQSPTPRPKKSYSGLRSPTRCVSEMLVIQHRERRERSRSRRKQHLLPPCVDEPELLF
SEGPSTSRWAAELPFPADSSDPASQLSYSQEVGGPFKTPIKETLPISSTPSKSVLPRTPE
SWRLTPPAKVGGLDFSPVQTSQGASDPLPDPLGLMDLSTTPLQSAPPLESPQRLLSSE**PL**
DLISVPFGNSSPSDIDVPKPGSPEPQVSGLAANRSLTEGLVLDTMNDSLSKILLDISFPG
LDEDPLGPDNINWSQFIPELQ

Supplementary Figure 2. The amino acid sequences of FOXM1 contained a PLDLI motif

The full length of amino acid sequences of FOXM1 were used to scan the PXDLX motif. A PLDLI motif was identified in its C-terminal and was indicated as the red letters.

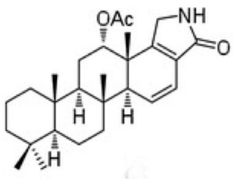

NSM00158

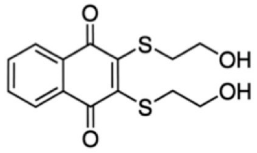

NSC95397

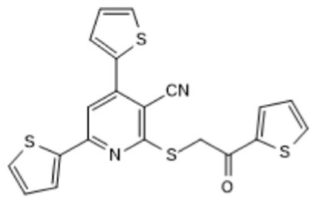

RCM1

Supplementary Figure 3. The chemical structures of NSM00158, NSC95397 and RCM1

The chemical structures of three small molecules, including NSM00158, NSC95397 and RCM1, were indicated.

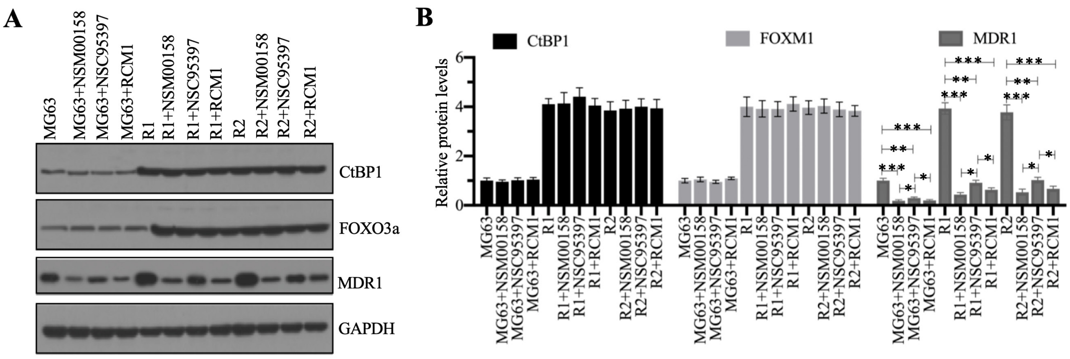

Supplementary Figure 4. The protein levels of CtBP1, FOXM1 and MDR1 in cells treated with different small molecules

(A) The protein band signals. Cells used in Figure 7A were subjected to western blotting assays to examine the protein levels of CtBP1, FOXM1, MDR1 and GAPDH (loading control). **(B)** The quantified protein levels. The protein band signals in (A) were quantified and normalized to their corresponding GAPDH. * $P < 0.05$, ** $P < 0.01$ and $^{***}P < 0.001$.

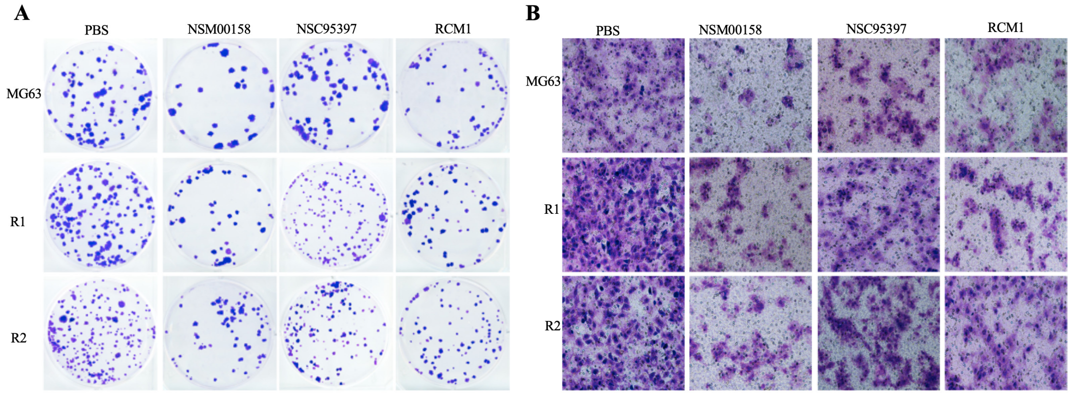

Supplementary Figure 5. The colony formation and cell migration assay results in cells treated with small molecules

(A) Colony formation assay. The same numbers of MG63, MG63-R1 and R2 cells were grown in sphere formation medium containing 2 μM NSM00158, 20 μM NSC95397 or 1 μM RCM1 at 37°C for 14 days. Colonies were stained with 0.1% crystal violet. **(B)** Cell migration assay. Cell suspension in serum-free DMEM containing 2 μM NSM00158, 20 μM NSC95397 or 1 μM RCM1 was subjected to Boyden chambers to determine cell migration. The migrated cells were stained with 0.1% crystal violet.

Supplementary Table-1. Primers used for vector construction

Vector	Forward (5'-3')	Reverse (5'-3')
pGL4.26-pMDR1^{WT}	GGGGTACCGATAGGGCTATAAACGT	CCGCTCGAGAAGTAGATTTCTTCATGTTC
pGL4.26-pMDR1^{Mut}	ATTGCAAAGGTTTATTATGAATTTC	GAAATTCATAATAAACCTTTGCAAT
pGADT7-CtBP1	CGGAATTCATGGGCAGCTCGCACTTG	CGGGATCCCTACAACTGGTCACTGGCGTGGT
pGBKT7-FOXM1	CCGGAATTCCATGAAAACTAGCCCCCGTC	CGCGGATCCCTACTGTAGCTCAGGAAT
pGBKT7-FOXM1$^{\triangle PLDLI}$	GCTCCTCAGTTCAGAATCCGTCCCCTTTGGCA	TGCCAAAGGGGACGGATTCTGAACTGAGGAGC
pCDNA3-2xFlag-CtBP1	CCCAAGCTTTGGGCAGCTCGCACTTG	CCGGAATTCCTACAACTGGTCACTGGCGTGGT
pCDNA3-MYC-FOXM1	CCCAAGCTTATGAAAACTAGCCCCCGTC	CCGGAATTCCCTACTGTAGCTCAGGAAT
pCDNA3-MYC-FOXM1$^{\triangle PLDLI}$	GCTCCTCAGTTCAGAATCCGTCCCCTTTGGCA	TGCCAAAGGGGACGGATTCTGAACTGAGGAGC

Supplementary Table-2. Primers used for RT-qPCR analyses

Gene	Forward	Reverse
CD34	GACCATGGAAGGCTTCCCAG	CACTTTGCTCCTGGGACAAGGC
CtBP1	TGGATGTGCACGAGTCGGAA	TGCGGATCTCCCGTGCCGCCT
MDR1	ATCCCAGTGCTTCAGGGACTG	TCAAGCAGCACTTTCCCTGCC
SOX2	AGTACTGGCGAACCATCTCTG	CCAACGGTGTCAACCTGCAT
TBX5	ACATGTAGGCAGGACTGTGA	TGACATCCAGTTTGGGTTGT
CDH1	ACTTGCAATGGGCAGCTATC	TCATAGTTCCGCTCTGTCT
BAX	AACTGATCAGAACCATCATG	AGATGGTCACGGTCTGCCACG
TIAM1	CTTGCTCAGTATGAGGAGCA	GCCTCTTTCCCGCTGACTGAT
NUPL1	AGCTCCTGTCGTTGGCTGCC	TGCATATTCATGTTGAAGTCC
FOXM1	CAGTTCAGACTATCAAGGAG	ATCCTCCCAGGAGTGAGATGA
β-Actin	ACTCCATCATGAAGTGTGAC	AGGAGCAATGATCTTGATCT

Supplementary Table-3. Differentially expressed genes in MG63-R1/R2 cells

Genes	MG63-1	MG63-2	MG63-3	R1-1	R1-2	R1-3	R2-1	R2-2	R2-3
CD34	-12.1	-10.3	-11.1	13.4	12.4	14.5	12.1	10.4	9.8
BCL2	-11.4	-10.1	-9.4	12.3	11.2	13.1	10.6	11.2	8.9
NANOG	-10.3	-9.4	-8.7	11.2	10.6	10.5	10.3	9.6	9.2
FOXM1	-10.2	-10.1	-8.9	10.9	10.2	9.5	8.9	8.7	8.4
TNFA	-9.4	-8.7	-9.2	10.3	9.5	10.3	8.2	8.3	8.1
CtBP1	-8.9	-8.3	-6.7	10.1	8.9	7.6	7.6	6.8	6.7
CD133	-8.5	-9.2	-7.8	9.8	8.3	9.2	7.2	7.6	7.2
Cyclin D1	-8	-6.8	-7.2	9.3	7.8	8.5	6.5	5.6	5.6
SIRT1	-7.6	-6.5	-5.6	9.1	7.2	7.2	6.1	7.8	7.3
MDR1	-7.2	-7.4	-5.3	8.5	6.5	6.7	5.6	6.7	5.4
CD44	-6.9	-5.8	-5.2	8.1	6.1	5.6	5.1	5.4	4.5
ATXN1	-6.4	-6.3	-6.5	7.6	5.7	5.4	4.6	5	4.3
LEF1	-6.2	-5.3	-5	6.7	5.4	6.7	4.3	4.5	6
GSK3B	-5.9	-5.2	-4.6	6.2	4.8	4.5	4.1	4	7.2
VEGF	-5.5	-4.7	-6	5.5	4.5	5.6	3.8	3.6	5.4
BIRC5	-5.2	-5.4	-4.3	4.9	4.3	4.7	3.6	3.4	3.4
TGFB1	-4.9	-4.2	-4.2	4.5	3.6	5.6	3.5	3.2	5.4
SOX2	-4.6	-4.6	-3.7	4.3	3.5	4.3	3.2	4.3	3.2
GDF6	-4.3	-5.1	-4.3	4	3.4	3.4	3	3.6	3.2
PLAT	-4	-4.3	-3.7	3.7	3.1	3.2	2.7	2.9	3
TBX5	-3.6	-3.6	-3.2	3.4	2.6	3.6	2.6	3.2	2.8
KIF4	-3.4	-3.2	-3	2.9	2.3	2.9	2.3	2.4	2.4
CDH1	11.1	10.4	9.5	-14.2	-13.1	-11.1	-10.2	-9.4	-9.2
PTEN	10.5	10.1	8.7	-13.2	-12.4	-10.2	-9.2	-8.6	-7.5
CDKN2A	10.1	10	7.4	-12.4	-12.1	-9.2	-7.6	-8.2	-8.7
BAX	9.4	8.7	8.7	-11.2	-11.2	-8.9	-7.2	-7.6	-6.5
DVL1	8.4	9.2	6.7	-10.4	-9.4	-7.8	-6.7	-8.4	-7.6
CTP2	8.2	7.8	6.2	-10.1	-7.8	-7.2	-6.2	-7	-5.5
CDKN1A	7.5	8.2	6.1	-9.3	-8.2	-8.5	-5.6	-8.3	-4.5

BIM	6.9	7	7.6	-8.6	-6.5	-8.1	-5.3	-6.5	-6.7
TIAM1	6.4	6.5	5.6	-8	-6.1	-6.5	-4.8	-5.4	-8.2
TNFSF10	6.3	6	5.3	-7.4	-5.4	-5.4	-7.6	-4.6	-5.3
CHEK1	5.6	5.6	8.2	-7	-7.2	-4.3	-5.4	-4.3	-4.4
CAPG	5.2	6.7	4.5	-6.5	-5.2	-6.5	-6.3	-6.5	-5.7
PUMA	4.8	5.4	5.4	-5.7	-4.5	-3.4	-5.3	-6.3	-4.3
KAI1	4.6	4.3	5.7	-5.2	-4	-4.6	-4.4	-3.5	-3.2
DACH1	4.3	4	3.2	-4.7	-6.5	-6	-3.7	-4.5	-6.5
GATA3	3.7	3.6	4.5	-4.3	-4.2	-3.2	-3.2	-5.6	-3.2
XPO4	3.5	5.5	3.6	-3.8	-3.7	-3.4	-5	-3.4	-6.1
GJB2	3.2	3	3.2	-3.4	-3.5	-2.8	-3.1	-3.2	-4.3
NUPL1	3	3.1	2.5	-3.2	-3	-3.2	-2.7	-3.5	-5
APAF1	2.8	2.5	2.4	-2.6	-2.7	-3	-3	-4	-4.3
